# Bioinspired core-shell nanofiber drug-delivery system modulates osteogenic and osteoclast activity for bone tissue regeneration

**DOI:** 10.1016/j.mtbio.2024.101088

**Published:** 2024-05-09

**Authors:** Shabnam Anjum, Yulin Wang, Yuan Xin, Xiao Li, Ting Li, Hengtong Zhang, Liang Quan, Ya Li, Dilip Kumar Arya, P.S. Rajinikanth, Qiang Ao

**Affiliations:** a Department of Tissue Engineering, School of Intelligent Medicine, China Medical University, Shenyang, Liaoning, 110122, China; b NMPA Key Laboratory for Quality Research and Control of Tissue Regenerative Biomaterial & Institute of Regulatory Science for Medical Device & National Engineering Research Centre for Biomaterials, Sichuan University, Chengdu, Sichuan, 610064, China; c Department of Laboratory Medicine, Shengjing Hospital of China Medical University, Shenyang, Liaoning, China; d Department of Pharmaceutical Sciences, Babasaheb Bhimrao Ambedkar University, Vidya Vihar, Raebareli Road, Lucknow, India

**Keywords:** Mussel-derived polydopamine, Osteoclastogenesis, PCL, PLGA, Bone regeneration, Core-shell nanofiber

## Abstract

Osteogenic-osteoclast coupling processes play a crucial role in bone regeneration. Recently, strategies that focus on multi-functionalized implant surfaces to enhance the healing of bone defects through the synergistic regulation of osteogenesis and osteoclastogenesis is still a challenging task in the field of bone tissue engineering. The aim of this study was to create a dual-drug release-based core-shell nanofibers with the intent of achieving a time-controlled release to facilitate bone regeneration. We fabricated core-shell P/PCL nanofibers using coaxial electrospinning, where alendronate (ALN) was incorporated into the core layer and hydroxyapatite (HA) into shell. The surface of the nanofiber construct was further modified with mussel-derived polydopamine (PDA) to induce hydrophilicity and enhance cell interactions. Surface characterizations confirmed the successful synthesis of PDA@PHA/PCL-ALN nanofibers endowed with excellent mechanical strength (20.02 ± 0.13 MPa) and hydrophilicity (22.56°), as well as the sustained sequential release of ALN and Ca ions. *In vitro* experiments demonstrated that PDA-functionalized core-shell PHA/PCL-ALN scaffolds possessed excellent cytocompatibility, enhanced cell adhesion and proliferation, alkaline phosphatase activity and osteogenesis-related genes. In addition to osteogenesis, the engineered scaffolds also significantly reduced osteoclastogenesis, such as tartrate-resistant acid phosphatase activity and osteoclastogenesis-related gene expression. After 12-week of implantation, it was observed that PDA@PHA/PCL-ALN nanofiber scaffolds, in a rat cranial defect model, significantly promoted bone repair and regeneration. Microcomputed tomography, histological examination, and immunohistochemical analysis collectively demonstrated that the PDA-functionalized core-shell PHA/PCL-ALN scaffolds exhibited exceptional osteogenesis-inducing and osteoclastogenesis-inhibiting effects. Finally, it may be concluded from our results that the bio-inspired surface-functionalized multifunctional, biomimetic and controlled release core-shell nanofiber provides a promising strategy to facilitate bone healing.

## Graphical abstract

**Figure:**
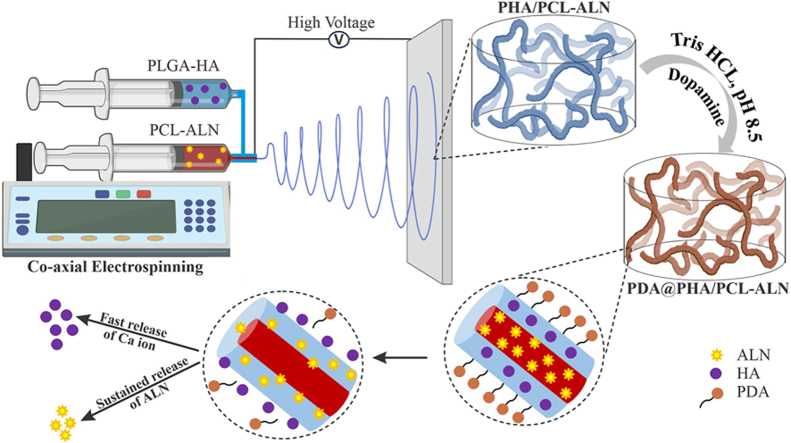

## 1 Introduction

Bone abnormalities are frequently observed in a clinical arena with serious social and economic impacts. Bone injuries generally exhibit an extended intrinsic healing duration, demanding a protracted duration for autologous regenerative mechanisms to reach culmination. The use of biomaterial-based bone grafts as alternatives for natural bone healing is common, and they have several benefits over previous grafting methods [1,2].

Ongoing research continues for an ideal bone graft alternative because allografts entail the risk of resorption, rejection, and disease transmission risks, while synthetic options like metals and ceramics are also used for bone tissue regeneration (BTR) [3,4]. However, the effective use of the aforementioned synthetic bone grafts is generally hindered by the low affinity of cell adhesion and inadequate or mismatched mechanical properties [5]. Bone remodeling is a highly complex biochemical reaction that involves not only osteogenesis (osteoblast-mediated bone formation) but also osteoclastogenesis (osteoclast-mediated bone resorption). Multiple cytokines play an important role in bone remodeling, which depends entirely on the balance between bone resorption and bone formation [6,7]. Creating a highly bioactive bone tissue engineering (BTE) scaffold capable of regulating the bone remodeling process and fostering bone regeneration poses a substantial challenge. Therefore, the use of a relatively simple yet highly effective strategy to regulate osteogenesis and osteoclastogenesis with a multifunctional and biocompatible scaffold is of paramount importance.

Bisphosphonates prevent bone resorption by selectively adsorbing to the mineral surfaces of bone, stimulating osteoblast activity, and suppressing osteoclastic activity [8]. Alendronate (ALN) is an FDA-approved nitrogen-containing bisphosphonate predominantly used as first-line treatment for osteoporosis [9]. The therapeutic potential of ALN as a bone resorption inhibitor has been demonstrated in several *in vitro* and *in vivo* experiments. ALN inhibits osteoclasts and enhances the expression of bone morphogenetic protein-2 (BMP2), a key factor responsible for osteogenesis and bone regeneration [10]. Conversely, the oral administration of ALN at high doses is likely to result in serious side effects, including jaw osteonecrosis and gastrointestinal ulcers. Additionally, high concentrations of ALN inhibit osteogenesis, while low concentration promotes osteoblast proliferation and osteogenic differentiation [11]. To solve this problem, controlled release of ALN with a scaffold would be an effective strategy.

While ALN possesses fascinating anti-osteoclastogenesis properties, it may not act as a standalone osteoinductive agent. The osteoconductive and osteoinductive qualities of calcium phosphate ceramics, also known as bioceramics, have made them a popular choice for BTR. Hydroxyapatite (HA), a type of bioceramic, naturally shares a great chemical and structural similarity with the minerals present in human bones. HA has gained popularity as a biomaterial for BTR owing to its biocompatibility, bioactivity, osteoconductivity and anti-inflammatory properties [12,13]. Additionally, HA integration can enhance the scaffold strength and cell activity while concurrently promoting angiogenesis and osteogenesis through osteoblast proliferation and differentiation [14]. Moreover, research has proved that HA exhibits high solubility in the physiological environment and can release nontoxic calcium (Ca) ions to stimulate blood vessel and bone regeneration by triggering calcium ion-sensing receptor signaling [15].

The FDA-approved biomedical polymers polycaprolactone (PCL) and poly lactic-*co*-glycolic acid (PLGA) have been used for controlled drug delivery, dressing, and tissue engineering in medicine [16]. PCL is a synthetic polymer with promising applications in bone tissue repair. Its unique properties include flexibility, a partially crystalline structure, and water repellency [17]. PCL is well known for its good biodegradability and mechanical properties; however, its hydrophobic nature limits its practical applications [18,19]. Single-phase PLGA is not suitable for BTR because of its dimensional instability and lack of osteoinductive and osteoconductive properties [20]. Shue et al. introduced an efficient technique to address the shrinking of PLGA. The results showed that adding size-stable PCL as the core fiber considerably enhanced the dimensional stability of the PLGA-based fibrous scaffold [21]. The hydrophobic surfaces of PCL and PLGA failed to promote cell attachment, prompting the combination of different biomaterials to create a suitable artificial bone scaffold. To address these issues and incorporate the properties of different materials into a single scaffold, we propose the use of the coaxial electrospinning technique. However, further surface modifications are needed to make it acceptable for cell attachment. Coaxial-structured nanofiber offers better controlled drug release than the single-nozzle and blend methods. The drug or protein contained within the core can be released slowly and remain effective for an extended period through the shell [[22], [23], [24]].

Different surface modification techniques have been developed to improve the surface adhesion of nanofiber scaffolds. Mussel-inspired molecule polydopamine (PDA) has been investigated for use as surface coating for biomedical implants because of its versatile adhesion properties, biocompatibility and biodegradability and its capacity to promote HA crystallization by offering more active sites, showing great potential in BTE [[25], [26], [27]]. PDA is also used as an immobilizing agent for different proteins on nanofiber scaffolds to enhance the loading rate as well as controlled and sustained release [28,29]. For example, Zhou et al. constructed calcium surface anchored collagen I-PLGA/PCL electrospun scaffolds (PP/COL-I-PDA-Ca). They achieved this by applying PDA coating to collagen I-incorporated PLGA/PCL substrates, which had been modified through the chelation of Ca^2+^. The results showed that the PDA-based Ca^2+^ chelation, COL-I incorporation, maintained 3D porous structure, and displayed good cytocompatibility. Additionally, ALP, OCN, OSX, RUNX2, and BMP-2 expression demonstrated that the combined effects of Ca^2+^ and collagen I boosted osteogenesis and bone cell differentiation [30].

**Scheme 1:**
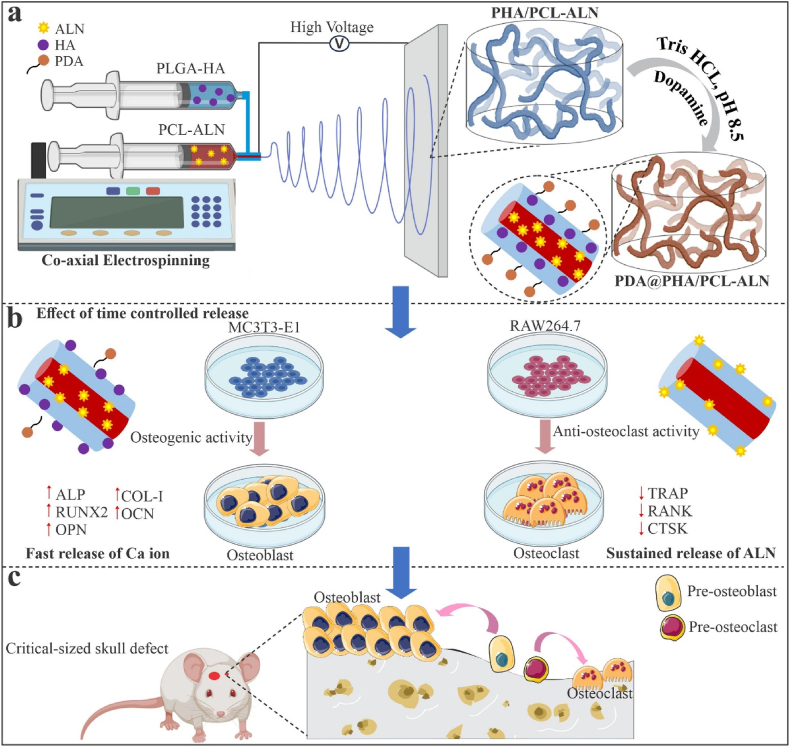
Schematic illustrations of the design and application of dual-delivery approach surface functionalized core-shell electrospun nanofibrous scaffold. **(a)** The PDA@PHA/PCL-ALN nanofiber was prepared by coaxial electrospinning method and surface functionalized by PDA. **(b)***In vitro* evaluation of surface functionalized PDA@PHA/PCL-ALN nanofibers in regulating osteogenesis and osteoclastogenesis. Initially, the surface modified and rapid release of HA loaded in the shell layer promote the recruitment and osteogenic differentiation of MC3T3-E1 cells, leading to increased bone regeneration. Subsequently, the delayed and sustained release of ALN in the core layer was designed to inhibit osteoclastogenesis and reduce bone resorption. **(c)***In vivo* evaluation of surface functionalized PDA@PHA/PCL-ALN nanofiber facilitates endogenous bone regeneration through synergistic approach of osteogenic stimulation and osteoclastic inhibition.

In this study, we fabricated ALN and HA-loaded core-shell nanofibrous scaffold using coaxial electrospinning and the surface of the nanofiber was functionalized with PDA to promote cell adhesion. The shell and core layer of nanofiber is composed of PLGA-HA (PHA) and PCL-ALN, respectively. As bone mesenchymal stem cells (MSCs) regulates the bone regeneration process *in vivo*, incorporating HA into the shell aims to promotes the osteogenic differentiation of MSCs, angiogenesis and enhance vascularized bone regeneration. Meanwhile, integrating ALN into the core layer could improve the sustained release of ALN and inhibit osteoclastic activity (Scheme 1). We investigated the physicochemical properties such as morphology, hydrophilicity, mechanical strength, *in vitro* ALN and Ca ion release, degradational behaviors of core-shell nanofibers. *In-vitro* and *in-vivo* studies were conducted to explore the effects of these scaffolds on cytocompatibility (i.e. cell attachment and proliferation), osteogenic differentiation, and osteoclastogenesis inhibition.

## 2 Materials and material processing

### 2.1 Materials

PLGA (LA/GA = 75:25, Mw = 105 kDa) was purchased from Shandong Medical Device Institute Co. Ltd. (P.R. China). PCL (Mn = 80 kDa), ALN (Mw = 325.12), 1,1,1,3,3,3-hexafluoro-2-isopropanol (HFIP), 2,2,2-trifluoroethanol (TFEA), Dopamine, Tris (hydroxymethyl aminomethane, Ninhydrin, 4′,6-diamidino-2-phenylindole (DAPI), and Rhodamine B were purchased from Macklin Biochemical Co. Ltd. (P.R. China). HA was previously synthesized in our lab. MC3T3-E1 and RAW264.7 cell lines were gift sample from professor Huang Zhongbing (Biomedical engineering collage; Sichuan university). All additional chemicals and reagents employed in the study were of analytical grade.

### 2.2 Fabrication of core-shell nanofibers by coaxial electrospinning

The core polymer solution, comprised of different concentrations (10 %, 12 %, and 15 % w/v), was prepared by dissolving PCL in TFEA. In parallel, the shell solution was prepared by dissolving different concentrations of PLGA (18 % and 20 % w/v) in HFIP. Electrospinning solution of PHA was prepared as follows: different concentrations of HA 4, 6, and 8 % (w/v) were ultrasonically dispersed in 10 mL of HFIP for 1 h. Then 20 % of PLGA was dissolved into the solution. PLGA was completely dissolved after vigorous stirring of the solution. For preparing PCL-ALN solution, 15 % of PCL was dissolved in 10 mL of TFEA and 1 % ALN was added and then stirred until complete dissolution. During electrospinning, the flow rates of the inner and outer solutions were 0.8 mL/h and 1.2 mL/h, respectively, with a tip-to-collector distance of 15 cm and an applied voltage of 22 kV. The coaxial electrospinning (TL-BM-300; Shenzhen TONGLI Micro Nano Technology Co. China) set-up was used in this study. Coaxial electrospinning was performed at 25 °C and 55 % relative humidity. To eliminate any residual organic solvent for future use, the electrospun scaffolds were vacuum-dried at room temperature.

### 2.3 Surface modification of nanofiber

PDA was applied onto the PHA/PCL-ALN scaffold via direct immersion coating method. For surface modification, electrospun PHA/PCL-ALN nanofibers were submerged in a dopamine solution (2 mg/mL in 10 mM Tris, pH 8.5) at 25 °C with mild shaking for 3 h. The color of the solution shifted from light pink to dark brown owing to the pH-induced self-polymerization of dopamine. The coated scaffold was subsequently rinsed three times with Milli-Q water to remove unbound dopamine molecules, resulting in PDA-coated PHA/PCL-ALN.

## 3 Results

### 3.1 Morphology of Core−Shell nanofibers

**Fig. 1:**
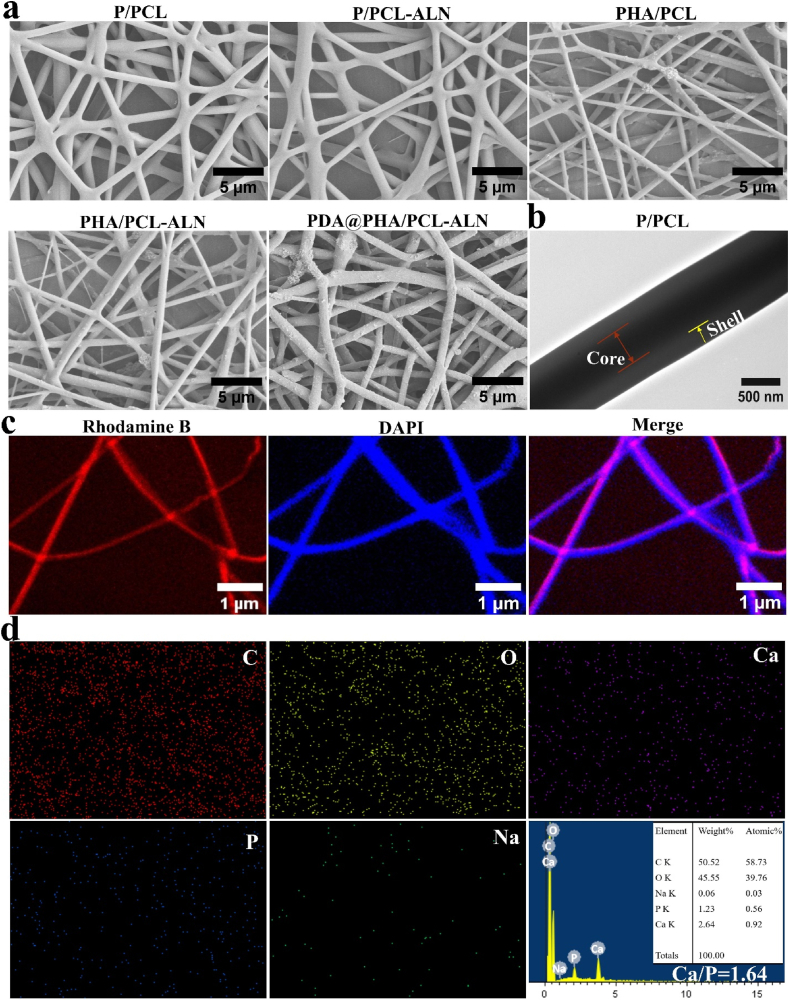
(a) Representative SEM images of different core-shell nanofibers; **(b)** TEM images clearly illustrate the distinctive core-shell structure in the P/PCL nanofibers; **(c)** CLSM images of the core-shell nanofibers with DAPI-labeled PLGA (shell) and rhodamine B-labeled PCL (core); **(d)** EDS elemental mapping and spectrum of PDA@PHA/PCL-ALN

During optimization, it was observed that the polymer concentration significantly impacts the formation of nanofibers, if the polymer concentration was not carefully controlled, the resultant nanofiber tends to exhibit numerous beads having no uniformity. Core-shell nanofibers were fabricated by optimizing the solution parameters. In the process of optimization, we found that the composition of 10%PCL-18%PLGA produced non-uniform nanofibers with beads. Increasing the concentration of PCL core solution from 10 % to 12 % (12%PCL-18%PLGA), nanofibers were formed with fewer beads (Fig. S1). Additionally, elevating both the core and shell solution concentrations to 15 % PCL and 20 % PLGA resulted in the formation of smoother and bead-free nanofibers (Fig. 1). For effective drug incorporation, we chose nanofibers produced from the 15 % PCL-20 % PLGA composition. However, the introduction of higher concentrations of HA into the polymer solution posed challenges during the electrospinning process, with potential HA aggregation within the fibres and reduced material mechanical effectiveness (Fig. S1). Therefore, we determined that 4 % HA concentration was the most suitable. It is clear from Fig. 1 that these nanofibers displayed a beadless morphology with random orientation. Therefore, five different scaffolds were constructed for further research in this study, denoted as P/PCL (placebo), P/PCL-ALN (ALN-loaded), PHA/PCL (HA-loaded), PHA/PCL-ALN (HA and ALN loaded), PDA@PHA/PCL-ALN (PDA surface functionalized HA and ALN loaded) core-shell nanofiber.

The morphology of the core-shell nanofibers is shown in Fig. 1a. All scaffolds had randomly aligned nanofibers and densely interconnected pores, mimicking the typical structure of the extracellular matrix (ECM). Additionally, from SEM images, the morphology of the HA nanoparticles was observed, indicating the successful fabrication and encapsulation of the HA within nanofibers. After PDA coating, the PDA@PHA/PCL-ALN scaffold showed a surface morphology similar to that of the PHA/PCL-ALN scaffold but was covered with PDA agglomerates (small aggregates occurred on the surface of the nanofibers). However, after the PDA coating, a very modest increase in average diameter was noticed. The histogram of the fiber diameter distribution showed that the addition of HA reduced the average diameter of the fibers (Fig. S2). Additionally, HA can increase electrical conductivity when incorporated as a mineral salt. Therefore, the polymer can be stretched completely in an electric field, which could account for the further reduction in the average fiber diameter with HA [20]. A slight decrease in diameter was also observed after loading ALN. Notably, PHA/PCL, PHA/PCL-ALN, and PDA@PHA/PCL-ALN exhibited significantly rougher morphologies than pure P/PCL because of the addition of HA into the nanofibers. It has been reported that roughened HA surfaces are more beneficial for cell attachment, proliferation, and subsequent osteogenic differentiation than smooth surfaces. The biological properties of biomaterials are determined by their structures. Micro-sized pores are formed between the interlaced fibers of the membrane, which can serve as a membrane barrier for the exchange of nutrients and waste material, thereby regulating the regeneration and repair of bone defects [20].

The structural integrity of core–shell P/PCL nanofiber along its axis was confirmed by TEM, as depicted in Fig. 1b. A clear core-shell structure is evident within P/PCL nanofiber, denoting its characteristic feature. To further verify the homogeneous dispersion of the solution within core in nanofibers, fluorescence staining was employed (Fig. 1c). DAPI (blue) was added into the PLGA solution and Rhodamine-B (red) into the PCL solution during nanofiber fabrication process. The distribution of core solution within nanofibers was further investigated using confocal laser scanning microscope (CLSM). The core-shell nanofibers displayed two distinct colors, as illustrated in Fig. 1c. The red dye was constrained in the middle of the nanofiber, whereas the blue dye was distributed around the PCL core. This outcome serves as evidence of the uniform distribution of the two constituents. The elemental composition of the scaffolds was determined through EDS elemental mapping, as shown in Fig. 1d and (Fig. S3). The PDA@PHA/PCL-ALN composite nanofiber is found to contain carbon (C), oxygen (O), phosphorus (P), sodium (Na), and Ca. This indicates the uniform distribution of ALN and HA within nanofibers. It exhibited that the two-step ultrasonic technique used in this work successfully blended the HA dispersion into the polymeric fibers, which is crucial for the mechanical strength and osteogenic behavior of the scaffolds. Further analysis of the EDS spectra revealed that PDA@PHA/PCL-ALN exhibits an atomic Ca/P ratio of 1.64, akin to the stoichiometric ratio observed in HA and in close proximity to the natural bone tissue ratio of 1.67.

### 3.2 Physiochemical properties

**Fig. 2:**
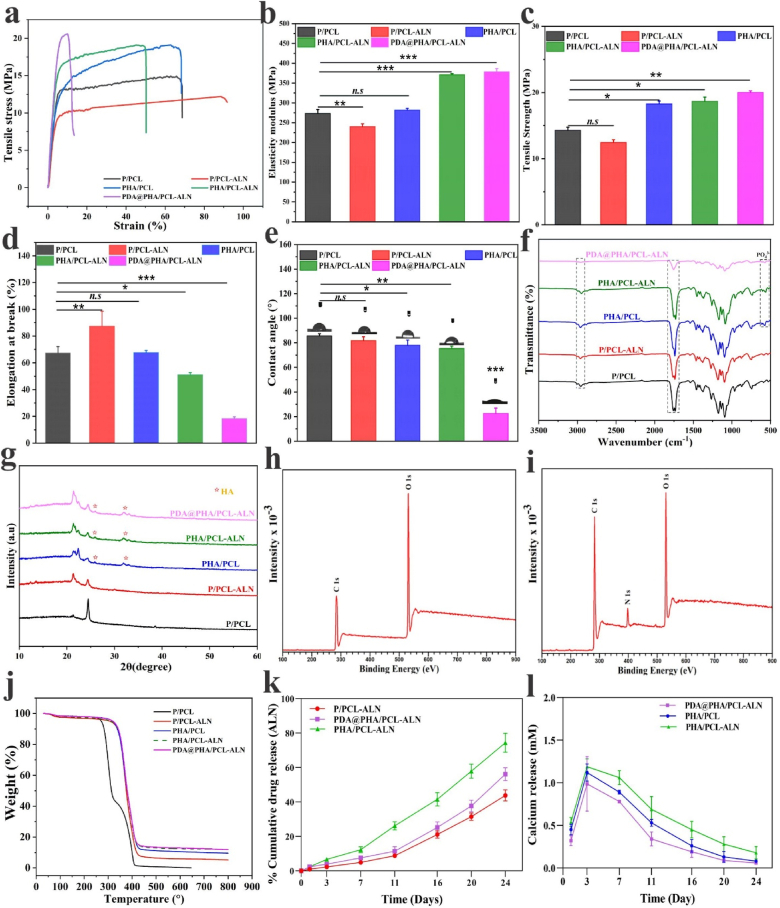
Different physicochemical characterization of developed non-functionalized (PHA/PCL, P/PCL-ALN and PHA/PCL-ALN) and PDA surface-functionalized (PDA@PHA/PCL-ALN) nanofiber scaffolds. (a) Typical strain-stress curves of different nanofiber; (b) Young's modulus; (c) Tensile strength measurement, and (d) Elongation at break; (e) Water contact angle; (f) FTIR spectra's; (g) XRD spectra's; (h–i) XPS analysis of PHA/PCL-ALN and PDA@PHA/PCL-ALN; (j) TGA analysis of different nanofibers under a N_2_ atmosphere; (k–l) Drug release behavior of ALN and Ca^2+^.

To develop a desirable biomaterial scaffold for BTR, the implanted material within bone defects must provide structural integrity while accommodating cell infiltration and fostering tissue regeneration. The mechanical characteristics of all scaffolds were examined using the standard stress–strain curves, as illustrated in Fig. 2a. The results demonstrated distinct levels of strength and resistance to deformation exhibited by each scaffold. In direct comparison to the P/PCL and P/PCL-ALN sample, the incorporation of HA and PDA coating within PHA/PCL, PHA/PCL-ALN, and PDA@PHA/PCL-ALN scaffolds notably augmented their maximum tensile strength. Notably, PDA@PHA/PCL-ALN possessed the maximum tensile strength among all groups, which may be due to the synergistic reinforcement of mineralized HA and PDA coating, consequently improving the matrix stiffness. Fig. 2b revealed that the Young's modulus of P/PCL exhibited a decrease from 273.28 ± 5.69 MPa to 239.89 ± 4.23 MPa upon incorporation of ALN (P/PCL-ALN). Subsequent addition of HA nanoparticles and PDA coating resulted in an increase in the Young's modulus of the electrospun nanofibers approximately (371.26 ± 1.47) for PHA/PCL-ALN and (378.70 ± 4.40) for PDA@PHA/PCL-ALN. Similarly, a noticeable variation in the tensile strength of PHA/PCL (18.28 ± 0.25 MPa), PHA/PCL-ALN (18.66 ± 0.37 MPa) and PDA@PHA/PCL-ALN (20.02 ± 0.13 MPa) was observed, as depicted in Fig. 2c. The observed values were significantly higher compared to those of P/PCL, with (14.29 ± 0.26 MPa) and P/PCL-ALN (12.44 ± 0.24 MPa). Moreover, Fig. 2d illustrated that P/PCL-ALN membranes exhibited a greater elongation at break. Notably, there was no significant difference detected in the elastic modulus and elongation at break of PHA/PCL compared to P/PCL. It is crucial to note that research indicates the elastic modulus and tensile strength of human cancellous bone span from 0.05 to 0.5 GPa and 1–20 MPa, respectively, contingent upon the apparent density [31]. The application of PDA coating on the scaffold demonstrates the requisite modulus and tensile strength akin to that of natural cancellous bone. It has been demonstrated by Shin et al. that the polymerization of PDA on the surfaces of PCL and PLA copolymers resulted in an increase in the polymer modulus of elasticity [32]. The PDA coating on the nanofiber functioned as an adhesive to enhance the interconnectivity and adhesion among the layers [33]. Previous study reported that the organic–inorganic integrity and homogeneous distribution of HA are beneficial for enhancing the mechanical characteristics of tissue engineering scaffolds [34]. Additionally, embedment of HA nanoparticles increased the mechanical strength of the mineralized scaffolds. This supported the microenvironment within the repaired bone defects and promoted the growth of new bone tissue [35]. The above findings confirmed the successful incorporation of ALN and HA into the core-shell P/PCL nanofiber, and resulting in the anticipated physicochemical properties of modified PDA@PHA/PCL-ALN scaffold. Thus, PDA-coated nanofibers could be ideal for non-load-bearing environments, such as cranial bone regeneration.

To determine the hydrophilicity of the nanofibrous scaffold, static water contact angle (CA) measurements were performed. As shown in Fig. 2e, the pure P/PCL core-shell nanofiber exhibited a water CA of 85.63°, demonstrating its hydrophobic nature. The addition of ALN did not change CA of the coaxial scaffold, but a significant decrease was observed in the scaffold (77.96°) PHA/PCL and (75.46°) PHA/PCL-ALN. The incorporation of HA nanoparticles enhanced the hydrophilicity of nanofiber because of hydrophilic P–OH group present on the surface of HA [36]. Notably, after PDA coating, the hydrophilicity of nanofibrous scaffold was enhanced and water CA of PDA@PHA/PCL-ALN was observed to be 22. 56°. This was attributed to the presence of hydrophilic amino and carboxyl-functional groups in PDA molecules. The surface wettability of the materials plays an important role in their interaction with cells. It has been documented in the literature that scaffolds with a CA between 5° and 40° are more conducive to cell attachment, proliferation, and differentiation *in vitro* as well as efficient penetration of host cells *in vivo* [37]. Consequently, the super-hydrophilic properties of PDA@PHA/PCL-ALN nanofibers were anticipated to positively impact BTR both *in vitro* and *in vivo*.

The functional groups present within the scaffolds were characterized using FTIR spectroscopy, as shown in Fig. 2f. The absorption peaks observed at 1759 and 1732 cm^−1^ were attributed to the free ester-based functional group of PLGA, and conjugated ester-based group of PCL, respectively [38,39]. Additionally, the spectrum exhibited a strong band near 1175 and 1090 cm^−1^ due to the presence of C–*O*–C group within the structure. The multiple peaks detected within the range of 1266-965 cm^−1^ were identified as C–*O*–C vibration mode. Upon the addition of ALN, no distinctive additional peaks associated with ALN were detected within these scaffolds, likely due to lower concentration of ALN. Notably, the FTIR spectra revealed the typical peaks of crystalline phosphate (PO_4_^3−^) at 613 and 523 cm^−1^, suggesting the presence of HA in the sample, (PHA/PCL, PHA/PCL-ALN, and PDA@PHA/PCL-ALN). Moreover, peaks at 2955 and 2880 cm^−1^ were identified as C–H stretching vibration in benzene methyl and methylene groups, respectively [40]. After the PDA coating, a reduction in the intensity of peaks related to methyl and methylene group was observed.

Then, X-ray diffraction (XRD) analysis was performed to discern the phase composition inherent within the nanofibers. The discernible peaks representative of the semicrystalline PCL (21.29° and 24.44°, respectively) and the HA phase peak (indicated by the symbol) were observed within the composite scaffolds Fig. 2g. Notably, in the case of polymer composites, with the addition of ALN, HA and PDA, there was an attenuation observed in the intensity of peaks corresponding to P/PCL-ALN, PHA/PCL, PHA/PCL-ALN and PDA@PHA/PCL-ALN. This reduction in intensity was noted without any discernible shift in peak position. This phenomenon could potentially be attributed to the diffraction characteristics exhibited by ceramic crystals or the potential overlap between the diffraction patterns of HA and ALN.

Further, X-rayphotoelectron spectroscopy (XPS) analysis was used to verify the surface modification of nanofibers. All the samples exhibited the elemental peaks of C 1s and O 1s (Fig. 2h and i). In contrast, a new nitrogen (N 1s) signal was discovered in PDA@PHA/PCL-ALN electrospun scaffolds, whereas, the absence of any detectable nitrogen peak was observed in PHA/PCL-ALN scaffold. The emergence of nitrogen (N 1s peak) at 398.52 eV indicates that the PDA coating was successfully deposited on the scaffold's surface.

The thermal stability of the different scaffolds was assessed via TGA. Fig. 2j demonstrates a marginal weight reduction (∼3.39 wt%) across all the samples between 55 and 150 °C, likely attributed to water evaporation. Notably, in the PHA/PCL sample, two distinct weight reduction phases were evident at 325 and 420 °C, signifying polymeric chain loss, culminating in complete material decomposition at 500 °C, displaying exceptional thermal stability. Additionally, the TGA curves showed analogous weight losses in PHA/PCL, PHA/PCL-ALN, and PDA@PHA/PCL-ALN, commencing around 285 °C and complete degradation at 650 °C. The thermal stability of the HA-loaded nanofibers was superior compared to other nanofibers. The thermal stability of nanofibers was improved by the addition of inorganic filler [41,42]. The TGA degradation profiles further demonstrated the effective addition of HA, which considerably improved the thermal stability of PHA/PCL, PHA/PCL-ALN and PDA@PHA/PCL-ALN scaffolds. In crafting biomaterial scaffolds for BTR, structural robustness and efficient load dispersion to surrounding bone tissues are imperative considerations.

Fig. 2k-l depict the release profiles of ALN and Ca^2+^ from various nanofiber scaffolds. Specifically, Fig. 2k illustrates the cumulative ALN concentrations released from P/PCL-ALN, PHA/PCL-ALN, and PDA@PHA/PCL-ALN scaffolds. The barrier effect inherent in the core–shell structure of the fibers was primarily responsible for the delayed release profile of P/PCL-ALN. After 24 days of incubation, only 44 % of the total ALN was cumulatively released from the P/PCL-ALN scaffold. In contrast, the cumulative quantity of ALN released from the PHA/PCL-ALN scaffold was 74.44 ± 5.4 % by day 24, which was much higher than the amounts released from the P/PCL-ALN scaffold (43.84 ± 3.19 %) and PDA@PHA/PCL-ALN (56.20 ± 3.7 %). PDA surface modification in PDA@PHA/PCL-ALN scaffolds potentially contributed to the lower ALN concentration released as compared to PHA/PCL-ALN. The core-shell nanofiber design sustains ALN release, potentially reducing its toxicity at higher doses. This controlled release is vital for optimizing ALN's therapeutic use, minimizing toxicity at elevated concentrations.

The presence of HA triggered a significant release of Ca^2+^. Across all the HA-loaded nanofiber groups, the initial three days showed a rapid burst release of Ca^2+^ (Fig. 2l). Additionally, the HA particles in shell layer of nanofibers such as PHA/PCL-ALN, PDA@PHA/PCL-ALN, and PHA/PCL became exposed and degraded over time. This resulted in slightly faster degradation compared to the P/PCL-ALN nanofiber. The initial burst release of Ca^2+^ from PDA@PHA/PCL-ALN was higher than that from ALN. This sequential release of ALN and Ca^2+^ holds promise for creating conducive environments for both reducing osteoclastic activity and inducing osteogenesis in both *in vitro* and *in vivo* study.

The degradation of biomaterials holds significant importance in tissue engineering and drug delivery. Ideally, materials should gradually break down after providing mechanical support for cell infiltration and tissue growth, facilitating uninterrupted tissue regeneration during bone healing. The degradation characteristics of P/PCL, P/PCL-ALN, PHA/PCL, PHA/PCL-ALN, and PDA@PHA/PCL-ALN are shown in Fig. S4. Initially, the fibrous scaffold exhibited the slow degradation within the initial 12 days (Fig. S4b). Over prolonged incubation periods, PDA@PHA/PCL-ALN, PHA/PCL-ALN, and PHA/PCL showed slightly higher weight losses compared to P/PCL-ALN and P/PCL. Notably, PDA@PHA/PCL-ALN displayed a comparatively faster degradation rate than other scaffolds, potentially attributed to hydrophilic surface being modified by PDA coating. Additionally, PDA undergoes oxidative biodegradation due to the presence of active oxygen species and free radicals, expediting the hydrolytic disintegration of the material and rendering it suitable for long-term implantation [36]. After 12 days, SEM was used to further analyze the degradation behavior (Fig. S4a). It was evident that the degradation caused changes in the fiber shape and breakage.

### 3.3 *In vitro* biological evaluation

#### 3.3.1 *In vitro* cytocompatibility

**Fig. 3:**
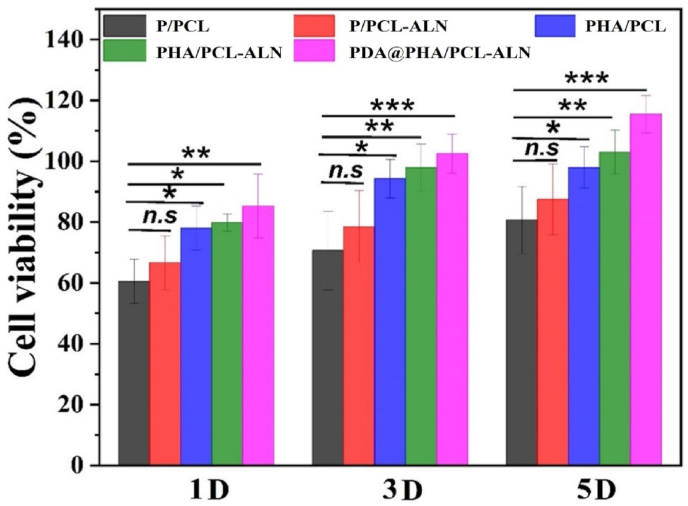
Cell viability was determined using the CCK-8 assay kit of various core-shell nanofiber scaffolds after 1, 3, and 5 days.

The cytocompatibility of functionalized scaffolds must be extensively evaluated due to the addition of multiple bioactive substances and/or drugs. Ensuring the non-toxic nature and favorable cytocompatibility of materials used in bone implants are essential prerequisites for effectively promoting BTR. In this study, MC3T3-E1 cells were cultured on various scaffolds including P/PCL, P/PCL-ALN, PHA/PCL, PHA/PCL-ALN, and PDA@PHA/PCL-ALN to investigate their cytocompatibility *in-vitro* via CCK-8 assay. The CCK-8 findings indicated uniform cell proliferation across the scaffolds, and the number of cells on PDA@PHA/PCL-ALN was significantly higher compared to other membranes on day 5 (Fig. 3). The advantageous microenvironment created by the combination of ALN, HA, and PDA coatings, fiber diameter, morphology, adequate mechanical properties, and superior hydrophilicity may be the cause of the beneficial cell behavior on the membranes. Additionally, incorporation of 4 % HA in scaffolds did not show any toxicity on MC3T3-E1 bone cells. The cell viability of MC3T3-E1 cells on core-shell nanofiber after PDA coating significantly enhanced on day 1, 3 and 5 as compared to uncoated scaffold. Previous researches have demonstrated that direct cell interactions on nanofibers provide a crucial modulatory role in cell destiny and function because of their ability to directly affect cell proliferation and differentiation [43]. Consequently, the nanofibers are speculated to support cell adhesion and proliferation.

**Fig. 4:**
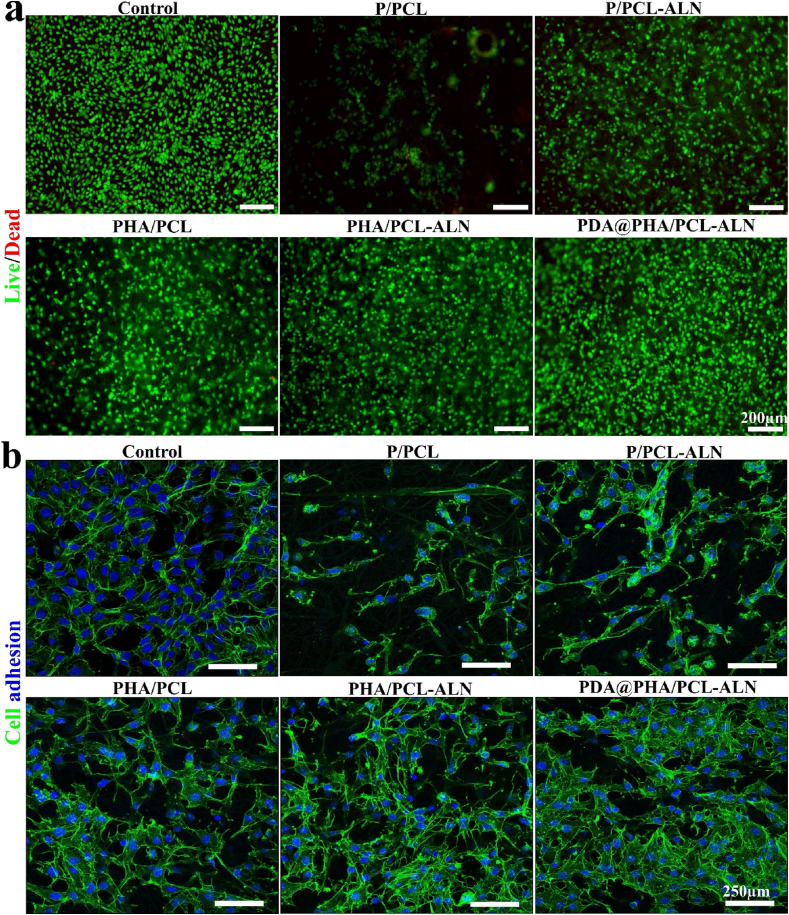
(a) Fluorescence microscopic images of live/dead staining of MC3T3-E1 cells cultured on different core-shell nanofiber scaffold's surfaces after 3 days. **(b)** Representative nucleus/cytoskeleton staining images of MC3T3-E1 cells cultured on different scaffolds after 3 days.

This was further supported by the findings of MC3T3-E1 cell's live/dead staining on the scaffold's surfaces, where the living cells were stained with (calcein AM) which produces green fluorescence when excited under blue laser (521 nm) and the dead cells were stained with (PI) which produces red color under blue laser excitation of 535 nm (Fig. 4a). The dead cells were rarely found in live/dead-stained images, and most MC3T3-E1 cells were alive and uniformly distributed, suggesting that all scaffolds had excellent cytocompatibility. After 3 days of live/dead assay, fluorescent images demonstrated that MC3T3-E1 cells were more widely distributed on PDA-modified PHA/PCL-ALN core-shell scaffolds compared to other non-modified nanofiber scaffolds. This corroborates previous findings that PDA coating increases the bioactivity of P/PCL composites.

The cellular responses to implanted biomaterials heavily rely on their adhesive capacity, crucial for subsequent cell behavior, morphogenesis, and eventual tissue responses. To further evaluate the cell adhesion and spreading morphology on distinct scaffolds was evaluated through phalloidin/DAPI staining, with observation via CLSM to assess the impact of these scaffolds on MC3T3-E1 cell cytoskeletal morphology, and the results are shown in Fig. 4b. Compared to the P/PCL and P/PCL-ALN scaffolds, cells provided a better spreading area on the PHA/PCL and PHA/PCL-ALN scaffolds after 3 days. Surface chemistry, roughness, and hydrophilicity of biomaterial scaffolds have a positive impact on osteoblast cell attachment. Previous research indicated that the rough surface of 10 % HA/PLGA stimulates cell attachment [44]. After PDA coating, PDA@PHA/PCL-ALN scaffolds exhibited a better cytoskeleton and healthier cell spreading areas, because of the linked porous structure, bone-like HA nanocrystals, and enhanced roughness and hydrophilicity on the surface. Therefore, PDA@PHA/PCL-ALN can promote cell stretching and adhesion across extended distances and is required for the proliferation and subsequent differentiation of MSCs for BTR [45]. Additionally, ECM proteins adsorbed on the substrate's surface, such as fibronectin, vitronectin, and other signaling molecules, significantly influence cell adhesion [46]. According to previous reports, PDA imparts positive charges to the material's surface, facilitating easier attachment to integrin receptors on the cell membrane, and exhibiting a special adhesion behavior [47].

#### 3.3.2 *In vitro* osteogenesis evaluation

Optimal bone implants must possess not only robust cytocompatibility but also exhibit favorable osteogenic activity [48]. Based on the aforementioned positive findings, the osteogenic differentiation of MC3T3-E1 cells induced by different scaffolds was assessed by measuring ALP activity, ARS staining, and the expression of genes associated with osteogenesis. The ALP activity of MC3T3-E1 cells was measured to evaluate their early osteogenic differentiation, and ARS was employed to detect calcium deposition, function as an indication of late-stage osteogenic differentiation.

**Fig. 5:**
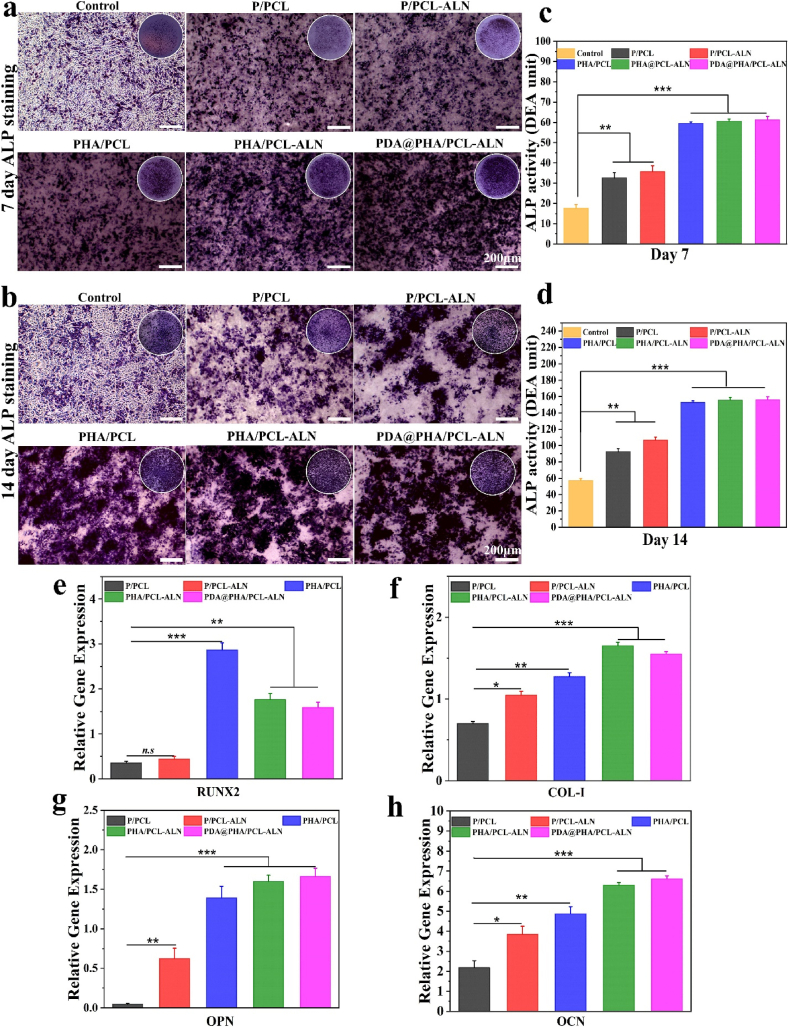
*In vitro* osteogenic activity of different core-shell nanofibers on MC3T3-E1 cells. **(a**–**b)** Representative images of ALP staining on different core-shell scaffolds after 7, and 14 days; **(c**–**d)** Quantitative evaluation of ALP activity; **(e**–**h)** Relative expression of different osteogenesis-related markers, including Runx2, Col-1, OPN, and OCN, after 7 days.

Fig. 5(a and b) displays higher staining density and distribution of ALP in PHA/PCL, PHA/PCL-ALN, and PDA@PHA/PCL-ALN groups compared to P/PCL and P/PCL-ALN groups after 7 and 14 days of incubation. The quantitative analysis was consistent with the corresponding staining Fig. 5(c and d). The *in-vitro* osteoblastic function of scaffolds containing HA was greater than that of scaffolds containing ALN (p < 0.001), suggesting that ALN alone had a limited impact on osteoblastic stimulation and differentiation. A possible explanation could be that ALN directly stimulates osteoclast differentiation and activation, but not osteoblasts [49,50]. Furthermore, we observed that the combination of HA and ALN provided higher ALP activity, indicating that the dual release of HA and ALN could enhance osteogenic differentiation of cells. After polydopamine coating, the results of mineralization analysis on 14th day indicated that the osteogenic efficiency of the PDA@PHA/PCL-ALN group was similar to PHA/PCL-ALN group and significantly better as compared to other groups, leading that the PDA coating had minimal function in early osteogenesis cell differentiation.

ARS staining was utilized to quantify calcium deposition across different composite scaffolds, with its interaction with Ca^2+^ in mineralized ECM and produces bright red staining. As shown in Fig. S6, after 14 and 21 days of culture, ARS staining showed that the calcification for PHA/PCL, PHA/PCL-ALN, and PDA@PHA/PCL-ALN was higher than that for P/PCL and P/PCL-ALN. This enhancement can be attributed to HA's osteoconductive properties and strong affinity for bone tissue. The integration of HA within the nanofiber shell layer facilitated calcium mineralization and enhanced osteogenic differentiation in MC3T3-E1 cells [51]. Notably, the osteogenic differentiation potential of MC3T3-E1 cells was significantly increased by PDA coating. The red calcium nodules in MC3T3-E1 cells on the PDA@PHA/PCL-ALN nanofibers were more abundant than that on the PHA/PCL-ALN scaffold. This aligns with earlier studies demonstrating the PDA coating's ability to concentrate Ca^2+^, potentially initiating HA nucleation and providing an efficient environment for osteogenic differentiation [52,53]. The observed increase in both early and late osteogenic differentiation demonstrated that the PDA@PHA/PCL-ALN nanofiber has a strong osteoinductive capacity and tremendous potential for BTR.

To further validate these results, the expression levels of osteogenesis-related genes i.e., Runx2, Col-1, OPN, and OCN were examined by qRT–PCR. Runx2 is a member of the runt homology domain family of transcription factors and serves as a crucial early marker in osteogenic differentiation, influencing the upregulation of Col-1 and OCN expression [54]. OPN, a glycosylated protein, is prominent in the middle and late stages of differentiation and plays an important role in bone remodeling and biomineralization [55]. OCN, a significant late-stage osteogenic marker secreted by osteoblasts, contributes a specific role in MSC differentiation and mineralization of the organic bone matrix [56]. As illustrated in Fig. 5e–h, a significant elevation in all osteogenesis-related genes were observed in MC3T3-E1 cells after 7 days of culture. In comparison with the P/PCL groups, the expression of these genes in the P/PCL-ALN, PHA/PCL, PHA/PCL-ALN, and PDA@PHA/PCL-ALN groups were significantly higher (p < 0.05, p < 0.01, and p < 0.001), which was consistent with the trends of ALP activity and ARS staining. Previous research has elucidated the mechanism through which Ca^2+^ from HA participate in various cellular physiological processes and facilitate the transmission of electrical impulses [57,58]. Furthermore, the effect of HA on stimulating osteogenic differentiation has been extensively examined [59]. Additionally, recent research found that HA-functionalized biomaterials could increase Runx2 expression and *in vitro* osteogenic differentiation [17,60]. The inclusion of HA within the shell layer provided favorable results by facilitating the continuous release of Ca^2+^. This promoted cellular mineralization and differentiation, and enhancing the formation of endogenic osteogenic components like Runx2 on PHA/PCL nanofibers *in vitro* (Fig. 5e). Jiang et al. [61] prepared a sandwich-like membrane and demonstrated that the incorporation of HA considerably increased cell osteogenic differentiation and mineralization. The Col-1 protein was abundant in the groups treated with PHA/PCL, PHA/PCL-ALN, and PDA@PHA/PCL-ALN scaffolds (Fig. 5f). In contrast, both P/PCL and P/PCL-ALN treated groups exhibited lower levels of Col-1proteins. Col-1, which plays a key role in bone remodeling, stands as the primary organic matrix component within bone tissue. Huanzhong et al. [62] prepared time-controlled release scaffolds via coaxial electrospinning, incorporating ALN within its core. Their results revealed that the dual delivery strategy inhibit osteoclast activity and induced osteogenic differentiation. An OPN is a secreted phosphoprotein within bones, plays a crucial role in bone homeostasis and metabolism. Interestingly, MC3T3-E1 cells exhibited significantly higher OPN expression on the PHA/PCL-ALN scaffold compared to the PHA/PCL scaffold, indicating an enhanced osteogenic effect facilitated by the prolonged release of ALN and Ca^2+^ (Fig. 5g). After PDA coating, OPN expression improved significantly in PDA@PHA/PCL-ALN in contrast to P/PCL, P/PCL-ALN, and PHA/PCL scaffolds. Conversely, no significant difference in the expression of OCN was observed between PDA@PHA/PCL-ALN and PHA/PCL-ALN scaffolds (Fig. 5h). In this study, we observed that PDA could slightly elevate the osteogenic differentiation of cells. The application of the proper PDA coating positively influenced cell differentiation during the excretion of proteins, particularly in the bone [63,64]. However, the combined use of ALN and HA demonstrated additive effects on osteogenesis. The dual delivery method is a suitable strategy for preventing the adverse effects of ALN by maintaining the dose within a safe range. These findings align with prior research [65,66]. In summary, the results exhibited that the design of coaxial fibers for a local sustained-release system promotes the osteogenic differentiation of pre-osteoblasts, verifying its desirable osteoinductive properties.

#### 3.3.3 *In vitro* osteoclastic evaluation

Bone healing relies not only on osteoblasts but also on osteoclasts, and controlling osteoclastogenesis is advantageous for effective bone regeneration. Therefore, it is important to consider not only the osteogenic capacity of biomaterials but also how they affect osteoclast differentiation when evaluating their bone regeneration properties. Osteoclasts, responsible for mineral resorption in the bone, are typically multinucleated large cells generated by the fusion of hematopoietic cells of the monocyte-macrophage lineage. The early stages of bone repair are delayed by the increased osteoclast activity. Specific stimuli such as receptor activator of nuclear factor κB ligand (RANKL) and M-CSF stimulate osteoclast development [67]. The formation of multinucleated giant cells in osteoclasts is caused by the activation of intracellular pathways, including nuclear factor κB (NF-κB) and nuclear factor of activated T cells c1 (NFATc1) [67]. Studies have demonstrated that ALN can inhibit osteoclast formation by suppressing the activation of the ERK1/2 and Akt pathways [68,69].

**Fig. 6:**
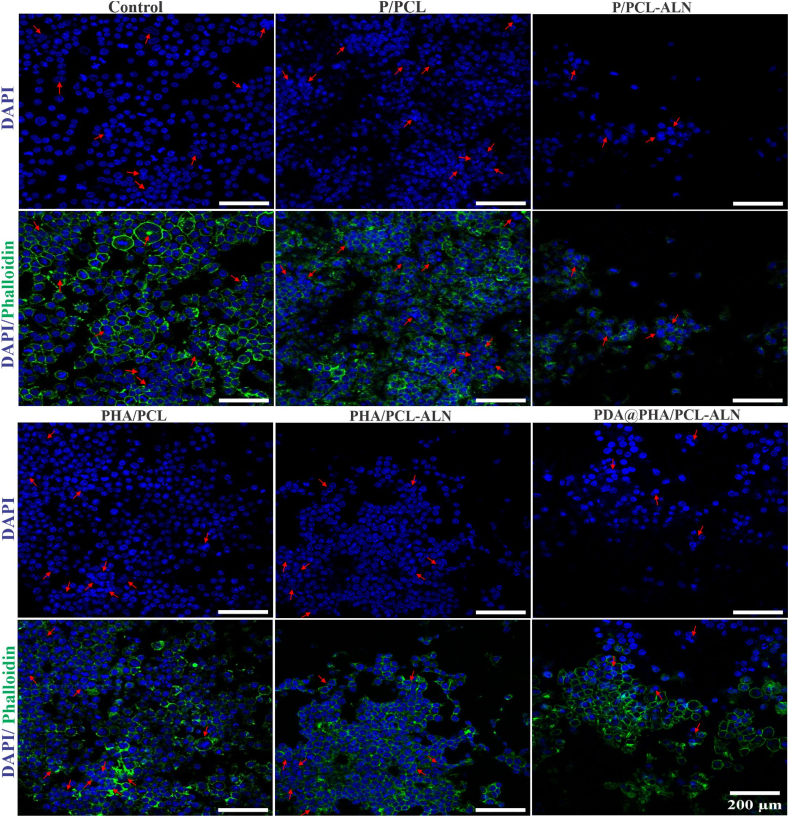
Representative nucleus/cytoskeleton staining images of RAW264.7 cells grown on different core-shell nanofibers. Cytoskeleton and cell nuclei were stained by Phalloidin (green) and DAPI (blue), respectively. Red arrows represent the formed osteoclasts. (For interpretation of the references to colour in this figure legend, the reader is referred to the Web version of this article.)

In this study, RAW264.7, a well-known model cell line for *in vitro* investigations of osteoclast was used as the primary model. To evaluate the effect of core-shell nanofibers on osteoclastic activity, we investigated the morphology of osteoclast differentiation. As shown in Fig. 6, multiple large multinucleated osteoclasts were observed on the surface of the P/PCL and PHA/PCL samples. Conversely, considerably less multinucleated osteoclasts were found on P/PCL-ALN, PHA/PCL-ALN, and PDA@PHA/PCL-ALN compared to the above two groups. The growth and maturity of functionally active osteoclasts is multistep complicated processes. These steps begin with the fusion of precursor cells to generate multinucleated cells, which results in multinucleated cells that undergo enormous cytoskeletal rearrangements, resulting in cell adhesion, cell polarization, and the formation of actin ring.

**Fig. 7:**
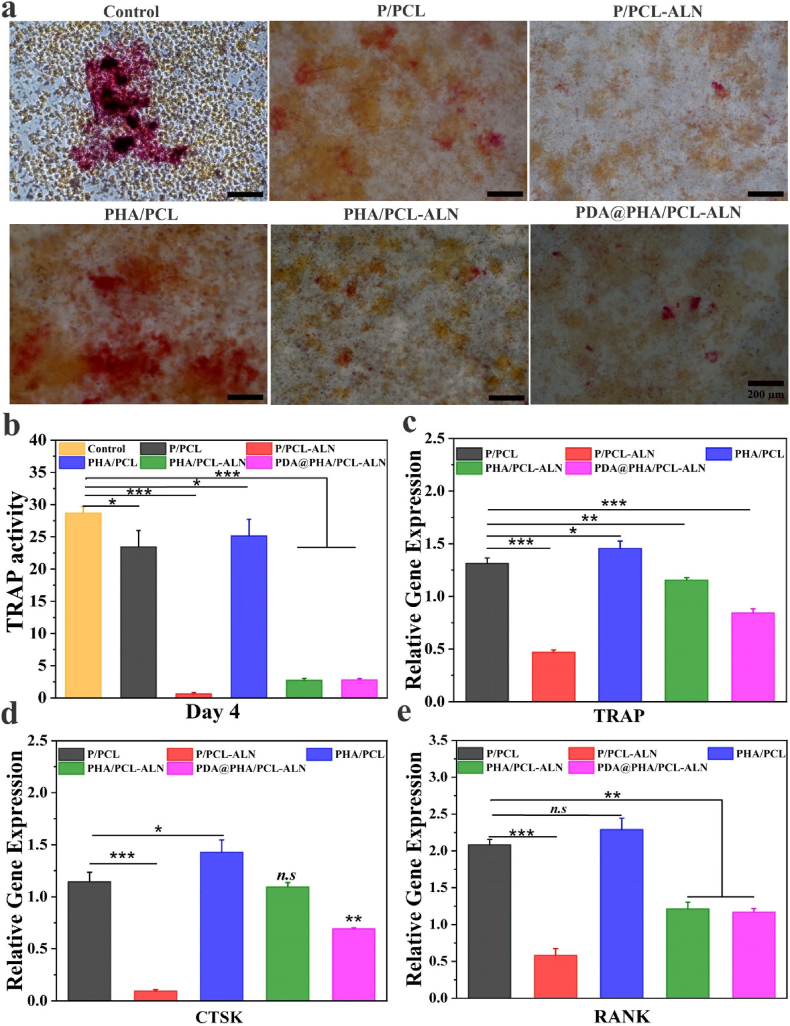
*In vitro* osteoclast activity of different core-shell nanofibers. **(a**–**b)** Representative TRAP staining images and enzyme activity of TRAP from RAW264.7 cells cultured on different core-shell nanofiber; **(c**–**e)** Gene expression of osteoclastogenesis-related markers, including TRAP, CTSK, and RANK in RAW264.7 cells after 4 days.

To acquire a better understanding of the role of ALN scaffolds on osteoclastogenesis, we employed TRAP staining and activity assessment. Furthermore, qRT-PCR was used to identify the expression of osteoclastogenesis-related genes, such as TRAP, cathepsin K (CTSK), and receptor activator of NF–B (RANK). In a classic experiment, osteoclast expression of the enzyme TRAP was stained to assess cell number and osteoclast resorptive activity *in vitro* [70]. The presence of TRAP-positive cells indicated the differentiation of macrophages into osteoclasts. After co-culturing RAW 264.7 cells with TRAP-positive multinucleated cells, we found that the quantity and size of these cells significantly decreased when exposed to P/PCL-ALN, PHA/PCL-ALN, and PDA@PHA/PCL-ALN nanofibers, demonstrating the inhibition of osteoclast development by the nanofiber, while significantly higher TRAP positive cell numbers were detected in the control, P/PCL, and PHA/PCL groups (Fig. 7a). Correspondingly, quantification of TRAP activity in cell lysate supported these findings (Fig. 7b). As illustrated in Fig. 7c-e, treatment with ALN-containing scaffolds significantly reduced TRAP, CTSK, and RANK expression (p < 0.01, p < 0.001), indicating reduced osteoclastic differentiation of RAW264.7 cells. Interestingly, the PDA-coated samples also inhibited osteoclast production compared to the P/PCL group, implying that PDA plays a role in the suppression of osteoclast maturation. Lufei et al. [71] showed that PDA-laced HA collagen material, without additional bioactive agents, could modify the cellular activity and transcriptome profile of osteoclasts. Overall, our findings support the assumption that PDA-coated PHA/PCL-ALN nanofibers can efficiently suppress osteoclastogenesis and enhance osteogenesis.

### *3.4* *In vivo* bone repair

The efficacy of bone regeneration and the biocompatibility of the engineered core-shell nanofiber scaffolds were assessed through critical-sized 4 mm diameter cranial bone defect models in male SD rats. Following precise suturing, the animals were housed in a controlled environment. Fig. S7(b) illustrates the surgical techniques used for implanting the materials. All animals were clinically healthy during the healing phase of the entire trial period. Images of the skull tissues collected from animals at 12 weeks are shown in Fig. S7 (c-d). The macroscopic integrity of the fibrous scaffolds was preserved, and none of them were completely disintegrated, indicating that the degradation rate of the coaxial fibrous scaffold aligned with bone regrowth. The nanofiber scaffolds exhibited excellent integration with adjacent tissues. At 12 weeks following implantation, the underlying area was predominantly occupied by bony tissues, and the residual material became thin and seamlessly interconnected with the surrounding tissues. It appears that the host bone accepted the newly created scaffold well, and there were no adverse effects on nearby tissues.

#### 3.4.1 Micro-CT analysis

**Fig. 8:**
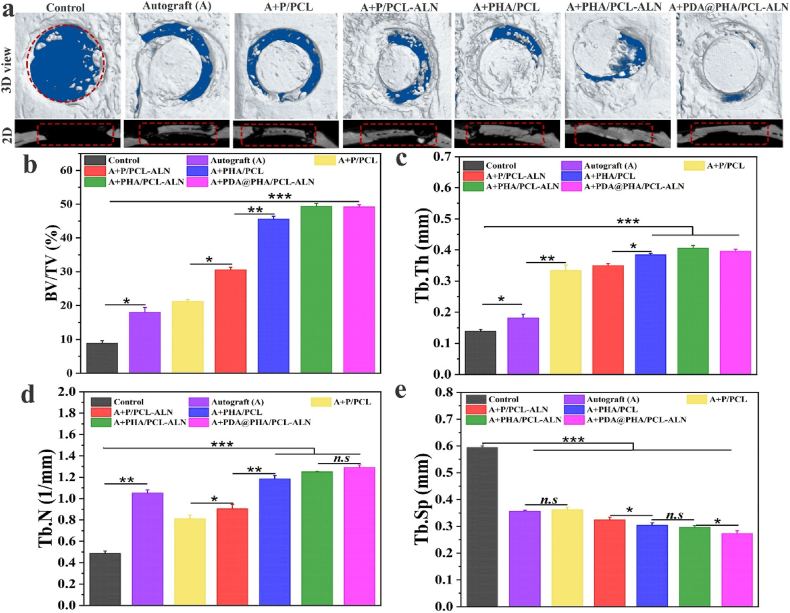
Representative micro-CT analysis of bone repair after 12 weeks of implantation of different core-shell nanofibers; (a) Coronal (3D) and transaxial (2D) views of cranial defects at 12 weeks after implantation. Red dotted lines indicate the initial boundary of the defect; (b–e) Quantitative analysis of BV/TV, Tb.Th, Tb.N, and Tb.Sp. (For interpretation of the references to colour in this figure legend, the reader is referred to the Web version of this article.)

At 12-weeks mark, micro-CT scanning was used to examine the development of new bone (Fig. 8a). The control groups showed predicted outcomes, with the majority of the defects remained unhealed and only a small quantity of freshly regenerated bone formed around the defect borders. Autograft (bone granules) exhibited superior outcomes compared to the control group. In contrast, the various nanofiber-treated bone defects displayed varying degrees of bone remodeling. The P/PCL nanofibers had a better repair effect than the control and autograft groups. This suggest that the porous and interconnected core-shell P/PCL-based scaffolds potentially function as templates facilitating adhesion and proliferation of bone progenitor cells in, thereby fostering mild bone regeneration. Additionally, we observed that the P/PCL-ALN and PHA/PCL groups had considerably greater bone regeneration capacity than the control, autograft, and P/PCL groups. More significantly, when compared to the other groups, the bone defects treated with PHA/PCL-ALN and surface-functionalized PDA@PHA/PCL-ALN displayed the most enhanced new bone formation area and increased thickness in comparison to other groups. This enhancement could potentially be attributed to the release of ALN and Ca^2+^ from PHA/PCL-ALN, in line with the outcomes observed in the *in vitro* studies. This improvement is also due to bioactive nature of HA and surface functionalization with PDA, which together provide sufficient mechanical support, help to promote osteogenic characteristics, regulate adhesion, proliferation, and differentiation, consequently expediting the process of bone regeneration.

The micro-CT data enabled morphometric analyses of the designated regions of interest (ROIs) to quantify bone repair efficacy, including the percentage of bone volume to total volume (BV/TV), trabecular thickness (Tb. Th), trabecular number (Tb. N), and trabecular separation (Tb. Sp), further confirming the above findings in Fig. 8(b–e). Overall, the PDA@PHA/PCL-ALN, PHA/PCL-ALN, and PHA/PCL exhibited notably higher BV/TV, Tb.Th, and Tb.N in comparison to other groups. However, Tb.Sp exhibited an opposite trend, with lower values detected in the PDA@PHA/PCL-ALN, PHA/PCL-ALN, and PHA/PCL groups. The volume fraction of the bone directly reflects the formation of new bone. The overall osteogenic effect of the PDA@PHA/PCL-ALN group was significantly greater than that of all the other groups. The synergistic microenvironment created by the combination of ALN, HA, and PDA may be responsible for the beneficial effects of bone repair. Remarkably, there was no statistically significant difference in the BV/TV between the PDA@PHA/PCL-ALN and PHA/PCL-ALN groups. Tb.N in the autograft group was higher than that in the P/PCL, P/PCL-ALN, and control groups. The PDA@PHA/PCL-ALN and PHA/PCL-ALN groups distinctly outperformed other groups in both BV/TV and trabecular spatial microarchitecture parameters after 12 weeks of implantation, which may be due to the increase in bone formation by osteoblasts and the decrease in bone resorption by osteoclasts on the trabecular surface. In addition, P/PCL and P/PCL-ALN scaffolds exhibited inadequate bone regeneration effects. This outcome indicates that in the absence of HA osteogenic induction cues, the P/PCL and P/PCL-ALN scaffolds only acted as a barrier to provide space for osteogenesis without sufficient osteoinductivity. However, this effect remained limited in the critical-sized cranial bone defect model of SD rats, aligning with findings from prior studies.

These findings suggest that the incorporation of HA, ALN, and PDA potentially promotes bone formation. Particularly, the PDA@PHA/PCL-ALN scaffold demonstrated notable synergistic effects, showcasing enhanced bone regeneration capabilities.

#### 3.4.2 Histopathology evaluation

**Fig. 9:**
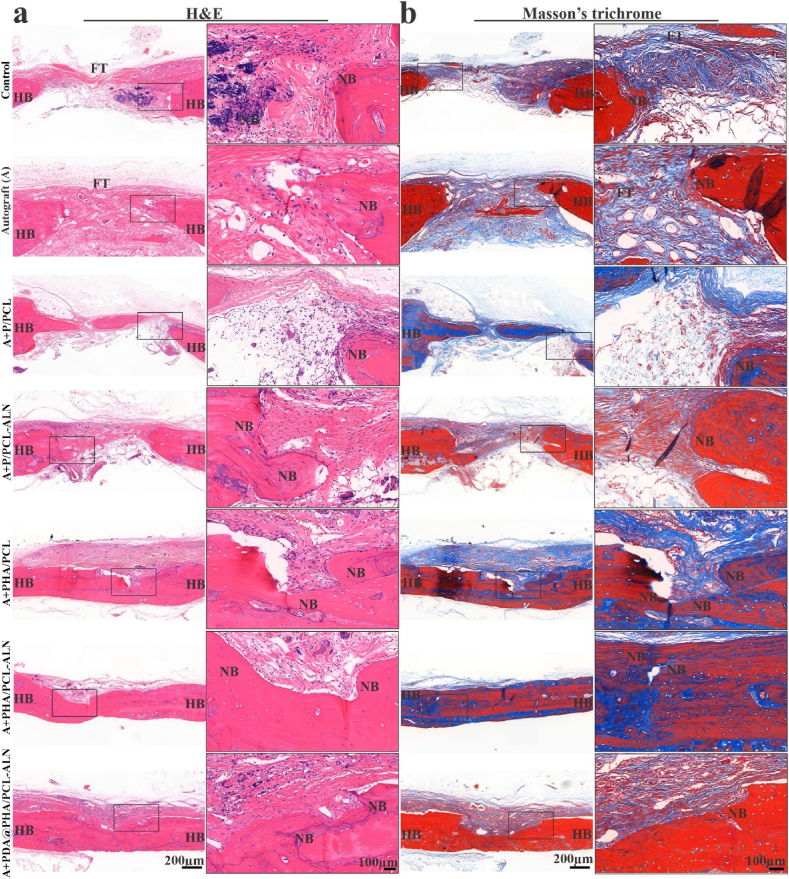
Histological evaluation for bone regeneration within the defect area exposed with scaffold 12-week post-implantation. (**a**) Representative H&E staining and (**b**) Masson's trichrome staining at 12 weeks after implantation. FT = fibrous tissue, HB = host bone, and NB = new bone.

After a 12-week implantation period, H&E and Masson's trichrome staining were carried out to further examine the pattern of bone growth within defect areas (Fig. 9). The findings of H&E staining are shown in Fig. 9a, and showed that only a thin layer of fibrous tissue developed in the control group whereas autograft (bone granules) showed better result compared to control group. This demonstrated that in a critical-sized cranial bone defect, new bone tissue growth is hardly possible without the aid of a scaffold. Consistent with the observations from micro-CT findings, there was evident new bone formation progressing from the periphery to the center of the defect in the PHA/PCL, PHA/PCL-ALN and PDA@PHA/PCL-ALN scaffold groups. This trend was particularly pronounced in the PDA@PHA/PCL-ALN scaffold group, indicating rapid Ca^2+^ release, sustained ALN release, and effects of PDA modification. These factors collectively facilitated the recruitment of MSCs, their subsequent osteogenic differentiation, and consequent mineralization within the scaffold.

Masson's trichrome staining was further used to find newly produced bone tissue and blood vessel within the defect site. Mature bones were appeared bright red in the Masson staining photographs, and immature bones as blue (Fig. 9b). The PDA@PHA/PCL-ALN group demonstrated better healing than other groups, which is consistent with the micro-CT results. These observations demonstrated that bone defect repair was enhanced and accelerated in the PDA@PHA/PCL-ALN, PHA/PCL-ALN, and PHA/PCL groups in contrast to the remaining groups. Additionally, the PDA@PHA/PCL-ALN and PHA/PCL-ALN scaffold groups demonstrated enhanced osteogenic potential, as both Masson's trichrome and H&E staining indicated more newly generated osteoblasts.

**Fig. 10:**
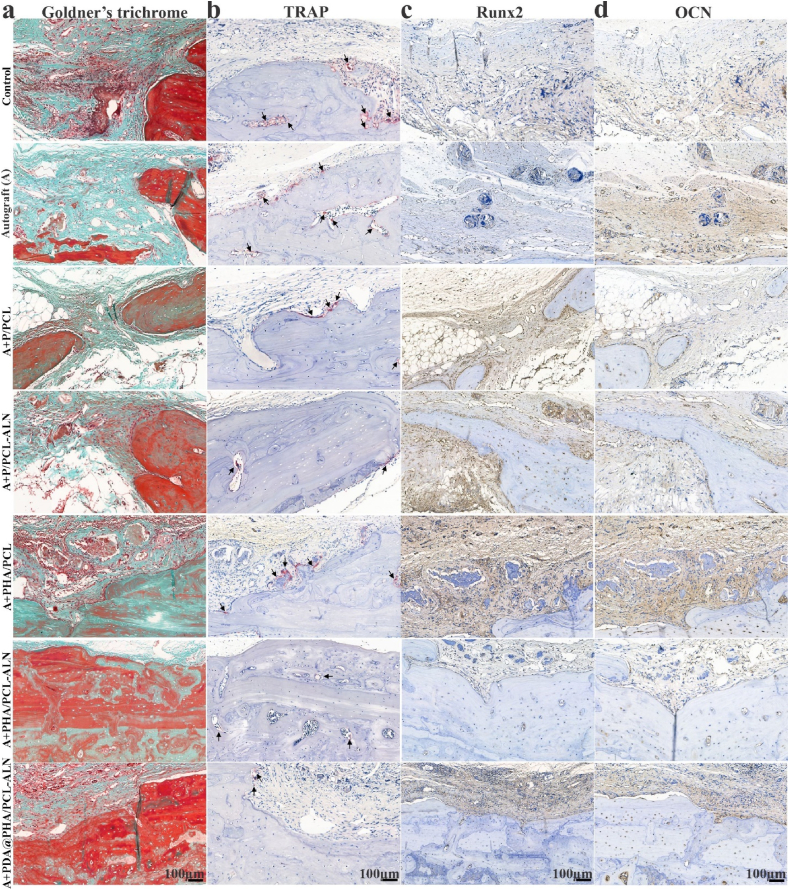
Histological evaluation and IHC staining for bone regeneration in the defect area with scaffold implantation. **(a)** Representative Goldner's trichrome staining; **(b)** TRAP staining at 12 weeks after implantation. Black arrow represents osteoclasts; **(c**–**d)** Representative IHC staining of Runx2 and OCN.

Goldner's trichrome and TRAP staining Fig. 10(a and b) were used to confirm *in vivo* bone mineralization and remodeling. As anticipated, the defect area within PHA/PCL-ALN and particularly the PDA@PHA/PCL-ALN group showed a significant amount of mineralized bone (dark green), along with a small number of TRAP-positive osteoclasts. This finding confirmed that the PDA@PHA/PCL-ALN scaffold promoted the maturation of new bone while suppressing bone resorption during bone repair. At 12 weeks after implantation, all other groups displayed minimal bone growth (identified as osteoid, stained orange/red), along with a notable population of osteoclasts. According to these findings, surface-functionalized core-shell PDA@PHA/PCL-ALN led to the dual ability to promote osteogenesis and inhibit osteoclastogenesis within the defect area. This dual effect synergistic accelerated both bone regeneration and remodeling. These findings were consistent with the *in vitro* biological performance findings.

#### 3.4.3 Immunohistochemistry

The decalcified bone tissue samples were subjected to immunohistochemical (IHC) staining to determine bone formation markers (Runx2 and OCN) within the defect region at the 12 weeks after implantation. This allowed for a deeper understanding of the underlying mechanism involved in scaffold-mediated osteogenesis and anti-osteoclastogenesis. Typically, OCN signifies a late stage of differentiation, while Runx2 is a hallmark of early osteogenic differentiation. IHC analysis revealed a notable increase in Runx2 and OCN expression at 12 weeks post-implantation, in the PHA/PCL, PHA/PCL-ALN, and PDA@PHA/PCL-ALN groups compared to the other groups (Fig. 10c and d). According to the aforementioned findings, our constructed PDA@PHA/PCL-ALN scaffold potentially facilitates in situ bone regeneration by strongly encouraging osteoblast development and subsequent mineralization of the bone matrix.

#### 3.4.4 Organ biocompatibility

The heart, liver, spleen, and kidney are among the primary organs that are removed after 12 weeks to be examined for cytotoxicity. The organs were divided into sections and stained with H&E for histopathological analysis, as depicted in Fig. S8. Remarkably, no significant pathological changes were evident in any of the groups, indicating the biocompatibility of the functionalized scaffolds without causing adverse local or systemic effects. The comparison of epithelial architecture in the kidneys between the control and experimental groups didn't reveal any notable differences. Similarly, both the follicular architecture and pulp structure of the spleen exhibited no signs of toxicity induced by the implanted scaffold. Moreover, the absence of haemorrhage in the heart suggests that the scaffold had no toxic effects in that organ. The morphological architecture of typical parenchymal cells of the liver did not exhibit noticeable differences from the control group. This comprehensive *in vivo* toxicity assessment indicated lack of post-surgical adverse effects of the implanted scaffolds on the test animal, emphasizes their safety and biocompatibility.

## 4 Discussion

Now a days, interest in finding a feasible strategy to facilitates bone regeneration has emerged. In this study, we successfully used coaxial electrospinning to synthesize ALN and HA enriched controlled dual delivery system (Scheme 1). SEM and TEM were employed to assess the nanofiber diameter and internal architecture. Confirmatory analysis of the core-shell structure was achieved via CLSM utilizing distinct dyes: Rhodamine for the core and DAPI for the shell. EDS with elemental mapping provided verification of ALN and HA incorporation within the core-shell structure. The C, O, P, Na, and Ca within the nanofibers confirmed the successful integration of both ALN and HA into the scaffolds. Additionally, SEM observations revealed a roughened surface morphology for PHA/PCL, PHA/PCL-ALN, and PDA@PHA/PCL-ALN, further supporting HA inclusion within the core-shell nanofibers. The surface coating of core-shell nanofibers with PDA was assessed via XPS. The presence of an (N 1s) signal exclusively in the PDA@PHA/PCL-ALN group verified the surface coating with PDA, as it was absent in all other groups. The superior mechanical properties of biomaterials are undeniably paramount for their application in BTR applications. Implant materials must exhibit high compressive strength to withstand the physiological loads experienced in the skeletal system. Studies have demonstrated that the mechanical strength of scaffolds, typically ranging from 8 to 20 MPa, can positively influence bone tissue in-growth. The tensile strength of the PDA@PHA/PCL-ALN scaffold emerged as the highest (20.02 ± 0.13 MPa) amongst all evaluated groups (PHA/PCL, PHA/PCL-ALN, PHA/PCL, and PHA/PCL-ALN). This suggests superior mechanical characteristics, potentially translating to improved functionality for BTR applications. Surface wettability of core-shell nanofibers is crucial for promoting cell attachment, proliferation, and differentiation. HA nanoparticle incorporation into core-shell scaffolds improved hydrophilicity due to surface-exposed hydrophilic P–OH groups. Notably, PDA coating further enhanced hydrophilicity, with PDA@PHA/PCL-ALN exhibiting CA of 22.56°, which is excellent for BTR. FTIR analysis corroborated the successful integration of ALN and HA within the core-shell scaffolds, along with PDA coating. The observed semicrystalline nature of the scaffolds further supported the incorporation of ALN and HA. TGA analysis demonstrated the exceptional thermal stability of all core-shell scaffolds between 55 and 150 °C with minimal weight loss, and addition of HA improved thermal stability of nanofibers. Additionally, core-shell scaffolds containing ALN exhibited slow and controlled release of ALN. Notably, PDA surface coating promoted even more sustained and controlled release of ALN, potentially mitigating ALN-associated cytotoxicity. A rapid release of Ca^2+^ ions was observed within 3 days for all core-shell nanofibers containing HA within their shell structure. This phenomenon is likely triggered due to the presence of HA. The *in-vitro* degradation of core-shell scaffolds play an important role in healing of bone defects. The biomaterials should possess slow degradation to facilitate uninterrupted tissue regeneration during bone healing. The developed core-shell scaffolds showed only around 60 % biodegradability after 4 weeks which is beneficial for BTR. Functionalization of scaffolds necessitates rigorous *in vitro* cytocompatibility evaluation due to the incorporation of bioactive moieties, a prerequisite for successful BTR. Consequently, the nanofibers are speculated to support cell adhesion and proliferation. All core-shell scaffolds demonstrated a statistically significant enhancement in cell viability (%) on days 1, 3, and 5. Notably, the PDA@PHA/PCL-ALN group exhibited the highest level of cell viability on MC3T3-E1 cells. This remarkable improvement in cell viability observed with the PDA@PHA/PCL-ALN group can be potentially attributed to the presence of the ALN, HA, and PDA coating on the core-shell nanofibers. Live/Dead assay provided compelling evidence for enhanced cellular adhesion and proliferation of MC3T3-E1 cells when treated with the various core-shell scaffolds. Notably, minimal dead cells were observed on the scaffolds and showed good cytocompatibility (Fig. 4a). Phalloidin/DAPI staining further corroborated these findings, demonstrating adequate adherence and proliferation of MC3T3-E1 cells across the different scaffolds, as visualized in Fig. 4b. *In-vitro* osteogenenic activity of all the core-shell scaffolds was assessed via ALP, ARS staining, and osteogenesis related gene expression. After 7 and 14 days, HA containing scaffolds (PHA/PCL, PHA/PCL-ALN, and PDA@PHA/PCL-ALN) exhibited significantly higher (p < 0.001) osteoblastic function compared to single ALN containing scaffolds (Fig. 5). These findings suggest that ALN may exert its effects primarily through the direct stimulation of osteoclast differentiation and activation, with minimal influence on osteoblastic activity. ARS staining was performed to quantify calcium deposition onto different scaffolds. Core-shell nanofiber scaffolds containing HA (PHA/PCL, PHA/PCL-ALN, and PDA@PHA/PCL-ALN) exhibited significantly higher calcium deposition compared to those without HA (Fig. 6). This enhanced deposition is likely due to the well-established osteogenic properties of HA, promoting bone formation on MC3T3-E1 cells. Notably, the PDA@PHA/PCL-ALN group displayed the highest level of calcium deposition, suggesting that the PDA coating further enhances the osteogenic potential of these scaffolds.

In addition to osteoblasts, osteoclasts have been found to play essential functions in bone healing; however, adequate reduction of osteoclastogenesis may be favoured for bone regeneration. Moreover, TRAP staining and enzyme activity revealed the significant decrease in osteoclast differentiation of RAW 264.7 cells treated with these scaffolds. Additionally, the significant downregulation of osteoclast related genes was observed in ALN loaded nanofiber treated groups. The scheduled temporal release of Ca^2+^ and ALN by core-shell nanofiber technology showed a remarkable improvement in osteoblast formation and osteoclast resorption. The *in-vivo* bone repair efficacy in cranial bone defect models in male SD rats was assessed by micro-CT analysis which showed higher BV/TV, Tb.Th, and Tb.N levels, clear indication of bone formation by increased number of osteoblast cells and decreased bone resorption by osteoclast cells on trabecular surface. The release kinetics of Ca^2+^ and ALN from these core-shell constructs have been shown to significantly enhance ALP activity, a well-established marker of osteoblast differentiation and intensify mineralized matrix deposition by osteoblasts as revealed by ARS staining, signifying augmented bone mineralization. *In-vivo* studies employing animal models have corroborated these findings, highlighting the ability of Ca^2+^ and ALN-releasing core-shell nanofibers to promote bone regeneration. Therefore, the meticulously controlled release of Ca^2+^ and ALN from core-shell scaffolds encompassing enhanced cell viability, differentiation, and mineralization, ultimately culminating in improved bone regeneration. Overall, our findings support the assumption that PDA-coated PHA/PCL-ALN nanofibers can efficiently work on BTR.

## 5 Conclusion

A novel surface-functionalized PDA@PHA/PCL-ALN nanofibrous scaffold with suitable physicochemical properties, enriched with ALN and HA was successfully created by combining core-shell electrospinning technology. The developed core-shell scaffolds showed uniform diameter distribution, appropriate mechanical strength and desired *in-vitro* and *in-vivo* degradation for BTR. This scaffold provides an advantageous milieu for MC3T3-E1 cell adhesion, proliferation, and differentiation and a potential multifunctional material for enhancing BTR. The scheduled temporal release of Ca^2+^ and ALN by core-shell nanofiber technology showed a remarkable improvement in osteoblast formation and osteoclast resorption. This study also emphasizes the significance of timing of medication release for the treatment of bone defects. Importantly, the *in vivo* evaluation of bone defect repair confirmed the aforementioned findings and showed that the scaffold promoted favorable osteogenesis while inhibiting osteoclastogenesis, eventually leading to strong bone regeneration. Therefore, this versatile implant is ideal for orthopedic application and this study also offers a green, simple, and affordable surface modification technique.

## CRediT authorship contribution statement

**Shabnam Anjum:** Writing – review & editing, Writing – original draft, Methodology, Investigation, Data curation, Conceptualization. **Yulin Wang:** Writing – review & editing, Software, Resources, Formal analysis. **Yuan Xin:** Writing – review & editing, Validation, Methodology, Formal analysis. **Xiao Li:** Writing – review & editing, Visualization, Validation, Software. **Ting Li:** Writing – review & editing, Validation, Funding acquisition, Formal analysis. **Hengtong Zhang:** Validation, Methodology, Investigation, Data curation. **Liang Quan:** Writing – review & editing, Validation, Software, Methodology. **Ya Li:** Validation, Methodology, Formal analysis. **Dilip Kumar Arya:** Writing – review & editing, Validation, Software, Formal analysis. **P.S. Rajinikanth:** Writing – review & editing, Validation, Methodology, Formal analysis. **Qiang Ao:** Writing – review & editing, Supervision, Resources, Project administration, Investigation, Funding acquisition, Conceptualization.

## Declaration of competing interest

The authors declare that they have no known competing financial interests or personal relationships that could have appeared to influence the work reported in this paper.

## Data Availability

Data will be made available on request.
