# Supplementary material for: Bioinspired core-shell nanofiber drug-delivery system modulates osteogenic and osteoclast activity for bone tissue regeneration
Source: Mater Today Bio. 2024 May 9;26:101088. doi: 10.1016/j.mtbio.2024.101088 (PMC11109009; doi:10.1016/j.mtbio.2024.101088)
Supplement: Multimedia component 1 [file mmc1.docx]

**Bioinspired core-shell nanofiber drug-delivery system modulates osteogenic and osteoclast activity for bone tissue regeneration**

Shabnam Anjum^1,2#^, Yulin Wang^2#^, Yuan Xin^2^, Xiao Li^1^, Ting Li^3,^ Hengtong Zhang^2^, Liang Quan^2^, Ya Li^2^, Dilip Kumar Arya^4^, P. S. Rajinikanth^4^, Qiang Ao^1,2*^

^1^Department of Tissue Engineering, School of Intelligent Medicine, China Medical University, Shenyang, Liaoning, 110122, China

^2^NMPA Key Laboratory for Quality Research and Control of Tissue Regenerative Biomaterial &amp; Institute of Regulatory Science for Medical Device &amp; National Engineering Research Centre for Biomaterials, Sichuan University, Chengdu, Sichuan, 610064, China

^3^Department of Laboratory Medicine, Shengjing Hospital of China Medical University, Shenyang, Liaoning, China

^4^Department of Pharmaceutical Sciences, Babasaheb Bhimrao Ambedkar University, Vidya Vihar, Raebareli Road, Lucknow

^#^These authors contributed equally to this work

*Corresponding Author Email: [aoqiang@scu.edu.cn](mailto:aoqiang@scu.edu.cn); aoqiang@tsinghua.edu.cn

**Characterization of Core-Shell Nanofibrous Scaffolds**

**Surface morphology**

SEM (S4800; Hitachi Japan) was used to investigate the surface morphologies of the nanofibrous scaffolds at different magnifications and an adjustable acceleration voltage. Before observation, the surfaces of the scaffold were sprayed with an Au-Pd layer through sputtering for a duration of 60 seconds. The obtained SEM images were analyzed at multiple locations by ImageJ software to determine the average diameters of the nanofiber scaffolds. Energy-dispersive X-ray spectroscopy (EDS) was used to characterize the elemental content and distribution inside the core-shell nanofiber scaffolds (S4800; Hitachi Japan). The observation of the core-shell structure of the nanofibers was conducted through Transmission Electron Microscopy (TEM; Tecnai G2 F20, FEI, USA) [1]. Core-shell nanofibers were deposited directly onto a copper grid with a carbon-coated layer and used for TEM imaging. To better distinguish the core and shell layers in the coaxial construction fibers, the electrospun solution was mixed with traces of fluorescent agents (DAPI in the shell solution and Rhodamine B in the core solution). Subsequently, the core-shell nanofibers were directly deposited onto a glass coverslip for visualization via Confocal Laser Scanning Microscopy (CLSM). The instrument employed for this analysis wasLSM880 Airyscan with STEDYCON; (Carl Zeiss, Germany). The mechanical properties of the core-shell nanofibrous scaffolds were evaluated employing a Universal Testing Machine (YG005A from Baien Instrument, China) with a 5cN capacity load cell at a crosshead speed of 8 mm/min. The evaluation of surface wettability, determining whether the nanofibers exhibit hydrophilic or hydrophobic properties, was conducted using a water contact angle instrument (model JC2000C1, POWEREACH, China). The analysis of the constituting functional groups within various core-shell nanofibers was performed using a Fourier Transform Infrared Spectroscopy (FTIR) spectrometer (NEXUS 670; NICOLET; USA). This spectroscopic technique allowed for the observation of structural alterations arising from electrospinning, blending, and coating processes etc. The FTIR scans were conducted utilizing a standard KBr crystal at room temperature (RT). Spectral data were acquired in transmission mode across a wavenumber range from 4000 to 400 cm^−1^. The assessment of the crystalline structure within the core-shell nanofiber scaffolds was conducted using an X-Ray Diffractometer (model DX-1000; PHILIPS USA). The thermal stability of various core-shell nanofibers was evaluated using a Thermo-Gravimetric Analysis (TGA) analyzer (TGA/DSC 2/1600-ThermoStar; METTLER TOLEDO Switzerland). Under a nitrogen atmosphere, the samples underwent controlled heating at a rate of 10°C per minute, covering a temperature range from 25°C to 800°C [2].

**X-ray Photoelectron Spectroscopy (XPS)**

Due to the ease of processing and data interpretation, XPS is frequently used for surface analysis. Photoelectrons were released from the sample surface when exposed to monochromatic X-ray radiation. Photoelectron binding energies were calculated using an electron energy analyzer. The elemental identification, chemical state, and amount of a given element can be deduced from the binding energy and intensity of the photoelectron peak. The thin-film structures on the surface layers, which reveal details about the surface modifications of polymers, were also revealed by XPS. XPS (AXIS Supra; Kratos UK) was used to analyze the surfaces of the core-shell nanofibers.

***In-vitro* Degradation**

The degradation profile of different core-shell nanofibers was investigated by monitoring weight loss at different time intervals. Initially, scaffolds measuring 1×1 cm² were precisely weighed (W_i_) and then placed individually into 5 mL plastic tubes, each containing 4 mL of phosphate buffer saline (PBS) (pH 7.4) solution. After that, these tubes were placed in a shaking incubator that was adjusted at 37°C temperature and 100 rpm speed. Weekly media changes were performed. At specific time intervals, the samples underwent drying until reaching a consistent weight (W_f_). This process allowed for the assessment of changes in weight over time, indicating the degradation pattern of the nanofibers in the PBS solution [3]. The % weight loss of core-shell nanofiber was calculated using the following formula:

$$\% Weight loss=\frac{W_{i}-W_{f}}{W_{i}}\times100$$

where *W_i_* and *W_f_* stand for the sample's original and final weight following deterioration.

***In-vitro* drug Release of Alendronate and Calcium ion**

To examine drug release from the formed core-shell nanofibers, the nanofiber mats were divided into 1×1 cm^2^ and submerged in a 10 mL solution of PBS (pH 7.4). The drug release was conducted at a controlled temperature of 37°C under agitation at 100 RPM within a thermostatic shaking incubator [4]. Successively, discrete 2 mL aliquots of the solutions were extracted at predefined intervals (1, 3, 7, 11, 16, 20, and 24 days), with equivalent volumes of fresh PBS introduced to preserve a consistent sink state. Quantification of alendronate release from distinct sets of nanofibers was executed utilizing UV-visible spectroscopy. Primarily, 1 mL of the liberated medium was mixed with 2.5 mL of a 0.2% ninhydrin solution and complemented by 0.5 mL of 0.05 M NaHCO_3_. This mixture was thoroughly mixed and subjected to a thermal treatment in a water bath, maintained at 95 ± 5°C for 35 minutes, resulting in a purple solution of 4 mL. Finally, the purple solution underwent dilution with deionized water, reaching a final volume of 5 mL, and the absorbance was measured at 568 nm.

Equivalent sampling techniques and time points were used in the calcium ion release experiments. A calcium colorimetric test kit (Elabscience, Cat.No.: E-BC-K103-M, China) was used to quantify calcium ion release. Calcium ions in the sample bonded to methyl thymol blue (MTB) in an alkaline solution, resulting in a blue complex, which may be used to quantify calcium release. Before measuring absorbance at 610 nm, the reaction system was incubated at RT and shielded from light for 5–10 min. The manufacturer's recommendations strictly followed this procedure.

***In-vitro* Hemocompatibility Test**

The hemocompatibility of the core-shell scaffolds were evaluated using freshly collected blood samples from the rats. Initially, 5 mL of blood was obtained into heparin-coated tubes and centrifuged at 3000 rpm at 4ºC to isolate red blood cells (RBCs) pellets. Post centrifugation, the supernatant was removed, and the RBC pellets were resuspended in 5 ml of PBS with a pH of 7.4. Another centrifugation at 3000 RPM was performed to further purify and isolate the RBCs. The resulting purified RBCs were diluted to make a final volume of 25 mL. Following this preparation, 0.5 mL aliquots of the RBCs suspension were dispensed into seven 1.5 mL tubes. Among these, two tubes containing PBS and distilled water served as negative and positive controls. The remaining five tubes were allocated for treatment with nanofibers e.g. P/PCL, P/PCL-ALN, PHA/PCL, PHA/PCL-ALN, and PDA@PHA/PCL-ALN each sized at 1×1 cm². Subsequently, all tubes, inclusive of controls and treated samples, underwent incubation at 37°C for a duration of 3 hours. Finally, all tubes were subjected to centrifugation at 3000 rpm for 10 minutes. 200 µL of each sample's supernatant was transferred to a 96-well plate and haemoglobin absorption at 545 nm was quantified. The %hemolysis of the control and samples were calculated using the following formula:

$$Hemolysis (\%) =\frac{{OD}_{Sample}-{OD}_{Negative Control}}{{OD}_{Positive Control}-{OD}_{Negative Control}}\times100$$

OD_Sample_, OD_Negative Control_, and OD_Positive Control_ represents the optical density (OD) of the samples, negative and positive control groups.

***In vitro* Cytocompatibility Evaluation**

**Cytotoxicity Assay (CCK-8 assay)**

The evaluation of MC3T3-E1 cell viability was conducted by employing the cell counting Kit-8 (CCK-8; KeyGEN BioTECH; China) following the manufacturer's protocols. Cells were seeded at a density of 1×10⁴ cells/well in triplicate onto different nanofiber in 96-well plates. Post-seeding, the cells were cultured for 1, 3, and 5 days. Following the respective culture durations, the media was removed and cells were washed with cold PBS with a pH of 7.4. After this, CCK-8 solution was added to each well, and the plates were incubated at 37°C for 2 hours. Subsequently, the OD at 450 nm was determined using a microplate reader (Thermo Fisher). The assessment of cell viability was calculated using the following formula:

$$Cell viability \left( \% \right)=\frac{{OD}_{Scaffold}}{{OD}_{Control}}\times100$$

**Live/Dead Staining**

MC3T3-E1 cells (1 × 10^4^ cells/well) were grown on top of the nanofiber scaffolds for 3 days in 48-well plate to further ascertain the cytocompatibility of the scaffolds. After 3 days of incubation, the culture media was removed carefully. The samples were rinsed thrice with cold PBS and stained for 20 min in the dark with 200µl of 1µg/ml acetoxymethyl ester of calcein (calcein AM) and propidium iodide (PI) staining solution (Solarbio, China). After incubation, the scaffolds were rinsed with cold PBS and fixed with 4% paraformaldehyde solution for 30 min. Images were acquired using an Inverted Fluorescence Microscope (Nikon).

**Cell Adhesion and Morphology of Nanofibrous Scaffold**

Cell adhesion was further investigated by staining actin cytoskeleton structures and visualizing the shape of the attached cells on top of core-shell nanofiber. The different core-shell nanofiber scaffolds were cut in appropriate size and immerged in 70% ethanol for 30 seconds followed by UV sterilization for 30 min. MC-3T3-E1 cells (1×10^5^ cells/well) were seeded on nanofiber scaffolds in 24-well plate. The cells were maintained in culture for a period of three days within a humidified incubator set at 37 °C and 5% CO_2_. After 3 days, culture media was removed and cells were washed three times with cold PBS (7.4, pH). The cells were fixed with 0.5 ml paraformaldehyde solution (4%) for 30 min. After fixation, cells were permeabilized with 0.5 ml of 0.1% Triton x-100 solution for 10 min. Following permeabilization, the scaffolds were stained with 500 µl of 5µg/ml DAPI (Solarbio; China) and 500 µl of 1µg/ml phalloidin (Solarbio; China) at RT for 10 min and 1 hr, respectively. Photographs were taken with an excitation filter of 405 nm (blue, DAPI) and 561 nm (green, phalloidin) using CLSM (LSM880 Airyscan with STEDYCON; Carl Zeiss, Germany).

***In-vitro* Evaluation of Osteogenic Ability****Alkaline Phosphatase Activity (ALP)**

ALP activity was measured to evaluate the capacity of MC3T3-E1 cells for early osteogenic differentiation. MC3T3-E1 cells at a density of 1×10^4^ cells per well were cultivated on nanofibrous scaffolds for 7 and 14 days. Proteins were extracted by adding a 1% Triton X-100 (Solarbio; China) solution. The resultant cell lysates were centrifuged for 10 min at 12,000 rpm to obtain the supernatant for the ALP assay. An Alkaline Phosphatase Assay Kit (Beyotime; China) was used to quantify the activity of ALP. A spectrophotometric microplate reader was used to determine OD at 520 nm.

For ALP staining, the adhered cells were fixed with 4% paraformaldehyde (Solarbio; China) and stained with a BICP/NBP ALP Stain Development Kit, according to the manufacturer's instructions (Beyotime; China) [5]. The samples were incubated at 37 ºC for 30 min and images were acquired using an Inverted Fluorescence Microscope (Nikon).

**Alizarin Red Staining (ARS)**

ARS staining employed to detect the calcium nodule development and matrix mineralization capacity of various nanofiber scaffold materials, which serve as indications of late-stage osteogenic differentiation. This parameter was measured after 14 and 21 days of incubation of MC3T3-E1 cells, seeded at a density of (1×10^4^ cells/cm^2^) on various nanofibers in 48-well plate. After incubation period, media was discarded and washed with 7.4 pH, PBS. Followed by this, the cells were fixed with 4% paraformaldehyde for 30 min.

ARS staining (Beyotime; China) solution was made by dissolving ARS in distilled water (DW) at a concentration of 2% (w/v) according to manufacture protocol. The fixed cells were stained with ARS, incubated for 40 min at RT and the extra ARS was removed with DW. An optical microscope was used to observe calcium deposition, and pictures were taken for examination.

**Osteogenic Gene Expression Assays**

Real-time PCR (RT-PCR) was used to assess the osteogenesis-related gene expression levels of MC3T3-E1 cells cultivated on various scaffolds, especially Collagen I (Col I), Osteopontin (OPN), Osteocalcin (OCN), and Runt-related transcription factor 2 (Runx2). Expression levels were computed using the 2^-ΔΔCT^ method. MC3T3-E1 cells (1 × 10^4^ cells/cm^2^) were grown on various nanofibers for 7 days. Total RNA was extracted using an RNAiso Kit (Beyotime; China). Then, using a PrimeScriptTM RT Reagent Kit (Beyotime; China), 2 µL of RNA was reverse-transcribed into single-stranded cDNA following the manufacturer’s protocol. The target genes Col I, OPN, OCN, and Runx2 were identified using SYBR Premix ExTM TaqII (Beyotime; China), with the primers listed in Table 1. The relative expression levels of the target genes were normalized to glyceraldehyde-3-phosphate dehydrogenase (GAPDH).

***In-vitro* Evaluation of Osteoclastic Ability**

**Cytoskeleton Staining**

RAW 264.7 cells (3×10^4^ cells/cm^2^) were cultivated in DMEM (high glucose) media supplemented with 10% fetal bovine serum (FBS) after being seeded onto various nanofiber scaffolds in 48-well plates. This medium was supplemented for four days with receptor activator of nuclear factor kappa-B ligand (RANKL) (50 ng/mL; PeproTech, China) and macrophage colony-stimulating factor (MCSF) (20 ng/mL; PeproTech, China). Next, cell nuclei and cytoskeleton were fixed with 4% paraformaldehyde, permeabilized and stained with 200 µl of 5µg/ml of DAPI and 200 µl of 1µg/ml of phalloidin, respectively. The stained cells were inspected using CLSM.

**Tartrate-Resistant Acid**[**Phosphatase**](https://www.sciencedirect.com/topics/materials-science/phosphatase) **(TRAP) Activity**

RAW 264.7 cells (3×10^4^ cells/cm^2^) were cultivated onto different nanofibers in 48-well plates and cultured in DMEM (high glucose) medium supplemented with 10% FBS. 50 ng/mL RANKL (PeproTech; China) and 20 ng/mL MCSF (PeproTech; China) were supplemented to this medium for 4 days. TRAP Assay Kit (Beyotime, China) was used to evaluate the TRAP activity of the RAW 264.7 cells following the manufacturer's instructions. The assessment was conducted using a spectrophotometric microplate reader, measuring the absorbance at 405 nm. Additionally, the activity of osteoclasts in each group was assessed by TRAP staining. Cells were fixed with 4% paraformaldehyde for 30 min and stained with TRAP dye (Beyotime; China) and incubated at 37°C for 30 minutes. After staining, the cells were properly rinsed with cold PBS (7.4). The cells were then photographed under a light microscope after rinsing with PBS (7.4) water to prevent excess staining. A single osteoclast-like cell was regarded as a TRAP-positive cell containing over three nuclei. **Osteoclastic Gene Expression Assays**

qRT-PCR was used to identify the expression of the osteoclastogenesis-related genes RANK, TRAP, and cathepsin K (CTSK), and the 2^-ΔΔCT^ technique was used to compute the expression. The gene expression level was normalized to the GAPDH.

Table 1 displays the primer sequences.

| **Target genes** | **Primer sequence** |
| --- | --- |
| **Col-1** | F:5′- AGAGGCATAAAGGGTCATCGTG-3′  R: 5′- AGACCGTTGAGTCCATCTTTGC-3′ |
| **OPN** | R: 5′-GCTGAAGCCTGACCCATCTC-3′  R: 5′-ATGGCTTTCATTGGAGTTGCTT-3′ |
| **OCN** | F: 5′-AGATTGTTGGGGCACAAG-3’  R: 5′-CCTTCAGCAGGGAAACCG-3′ |
| **Runx2** | F: 5′- GGAACCAAGAAGGCACAGACAG-3’  R: 5′- TGTCTGCCTGGGATCTGTAATCT-3′ |
| **RANK** | F: 5’- CGGCGTTTACTACAGGAAGGG -3’  R: 5’- CTTCTTGCTGACTGGAGGTTGC -3’ |
| **TRAP** | F: 5’- GTGACAAACAGTACCTCCACGATG -3’  R: 5’- GCCCTTGATTGGGTTCACAGT -3’ |
| **CTSK** | F: 5’- TTACTCCAGTCAAGAACCAGGGC-3’  R: 5’- GCCTCCACAGCCATAATTCTCA-3’ |
| **GAPDH** | F: 5’- CTGGAGAAACCTGCCAAGTATG-3’  R: 5’- GGTGGAAGAATGGGAGTTGCT -3’ |

***In-vivo* Experiments**

**Experimental Study on Repairing Skull Defects in Rats**

All animal surgeries were carried out in accordance with Sichuan University's Animal Ethical Committee. To evaluate the effectiveness of bone regeneration, we used a critical-sized skull defect model in Sprague-Dawley (SD) rats (n = 36, 200 gm weight). To exposed the cranial bone, the epicranium of the SD rats was shaved and anesthetized with pentobarbital sodium (30 mg/kg body weight). A saline-cooled trephine drill was used to create two critical-sized whole-thickness bone defects, each measuring 4 mm in diameter, on each side of the parietal bone midline. The bone blocks were scraped from the defects and collected for further use as bone granules. Each defect received different core-shell nanofiber therapy.

**Group 1; Control Group**

(a) Control group with nothing filled inside **(left side of skull defect)**

(b) Only autogenous bone granules were placed **(right side of skull defect)**

**Group 2**; **P/PCL**

(a) Defect was filled with autogenous bone granules (Autograft) (**Left)**

(b) Autogenous bone granules were implanted into the defect and PCL/P scaffold with a diameter of 8 mm was cover upon the implanted bone **(Right)**

**Group 3;** **PHA/PCL**

(a) Defect was filled with autogenous bone granules **(Left)**

(b) Autogenous bone granules were implanted into the defect and PHA/PCL scaffold with a diameter of 8 mm was cover upon the implanted bone **(Right)**

**Group 4; P/PCL-ALN**

(a) Defect was filled with autogenous bone granules **(Left)**

(b) Autogenous bone granules were implanted into the defect and P/PCL-ALN scaffold with a diameter of 8 mm was cover upon the implanted bone **(Right)**

**Group 5; PHA/PCL-ALN**

(a) Defect was filled with autogenous bone granules **(Left)**

(b) Autogenous bone granules were implanted into the defect and PHA/PCL-ALN scaffold with a diameter of 8 mm was cover upon the implanted bone **(Right)**

**Group 6; PDA@PHA/PCL-ALN**

(a) Defect was filled with autogenous bone granules **(Left)**

(b) Autogenous bone granules were implanted into the defect and PDA@PHA/PCL-ALN scaffold with a diameter of 8 mm was cover upon the implanted bone **(Right)**

The wound was meticulously stitched up with 4-0 nylon sutures after implantation. Cranial tissues were promptly collected for further radiographic and histological evaluations when the rats were sacrificed at 12 weeks by injection with an overdose of anesthetic.

**Imaging Observation (Micro-CT analysis)**

After implantation for 12 weeks, all the SD rats were euthanized and bone samples were fixed in 10% paraformaldehyde. In accordance with the established methodology, cranial samples were scanned using Bruker Micro-CT (Skyscan 1276 system, Germany). Under the conditions of 85 kV and 200 μA, and with scanning resolution of 10.154194 μm, 2D photographs of each specimen were captured. Following scanning, the 3D structure of the samples was recreated using NRecon software, with the following settings set: smoothing = 5, ring artifact correction = 25%, and beam hardening correction = 8. We set up the region of interest (ROI) as the 4 mm diameter bone drilling area to statistically measure new bone growth in the defect area. The parameters of bone regeneration, including the percent of bone volume (BV/TV), trabecular thickness (Tb.Th), trabecular number (Tb.N), and trabecular separation (Tb.Sp) to investigate bone formation in the region of interest were quantified using software.

**Histopathology Evaluation**

After micro-CT scanning, the collected cranial samples were decalcified in 10% EDTA. After being embedded in paraffin, the tissues were sliced and stained with hematoxylin-eosin (H&E) to visualize new bone growth. Newly developed bone tissue and blood arteries in the defect location were detected using Masson's trichrome staining. To assess bone remodeling and mineralization, deparaffinized sections were stained with TRAP and Goldner's trichrome. Furthermore, immunohistochemical staining of Runx2 and OCN, was carried out to analyze the expression of osteogenesis-related proteins. In brief, 5% bovine serum albumin (BSA) solution was used to block the deparaffinized sections, then incubated with primary antibodies against Runx2 and OCN as osteogenesis indicators at a 1:100 dilutions overnight at 4 °C. The major organs in each group were fixed with 10% paraformaldehyde, embedded in paraffin, cut into sections, and stained with H&E for histological analysis to investigate the long-term *in vivo* biosafety of the implanted scaffolds at 12 weeks after implantation.

**Statistical Analysis**

All experiment data were shown as mean±S.D.. The data from all experiments were evaluated using one-way ANOVA followed by [Dunnett's test](https://en.wikipedia.org/wiki/Dunnett%27s_test) selected to discern the significant differences between groups. Statistical analysis was conducted using Origin 2019 software. P-value less than 0.05 was considered statistically significant. *p < 0.05, **p < 0.01, ***p < 0.001, and n.s denotes no significant difference.

**Results**


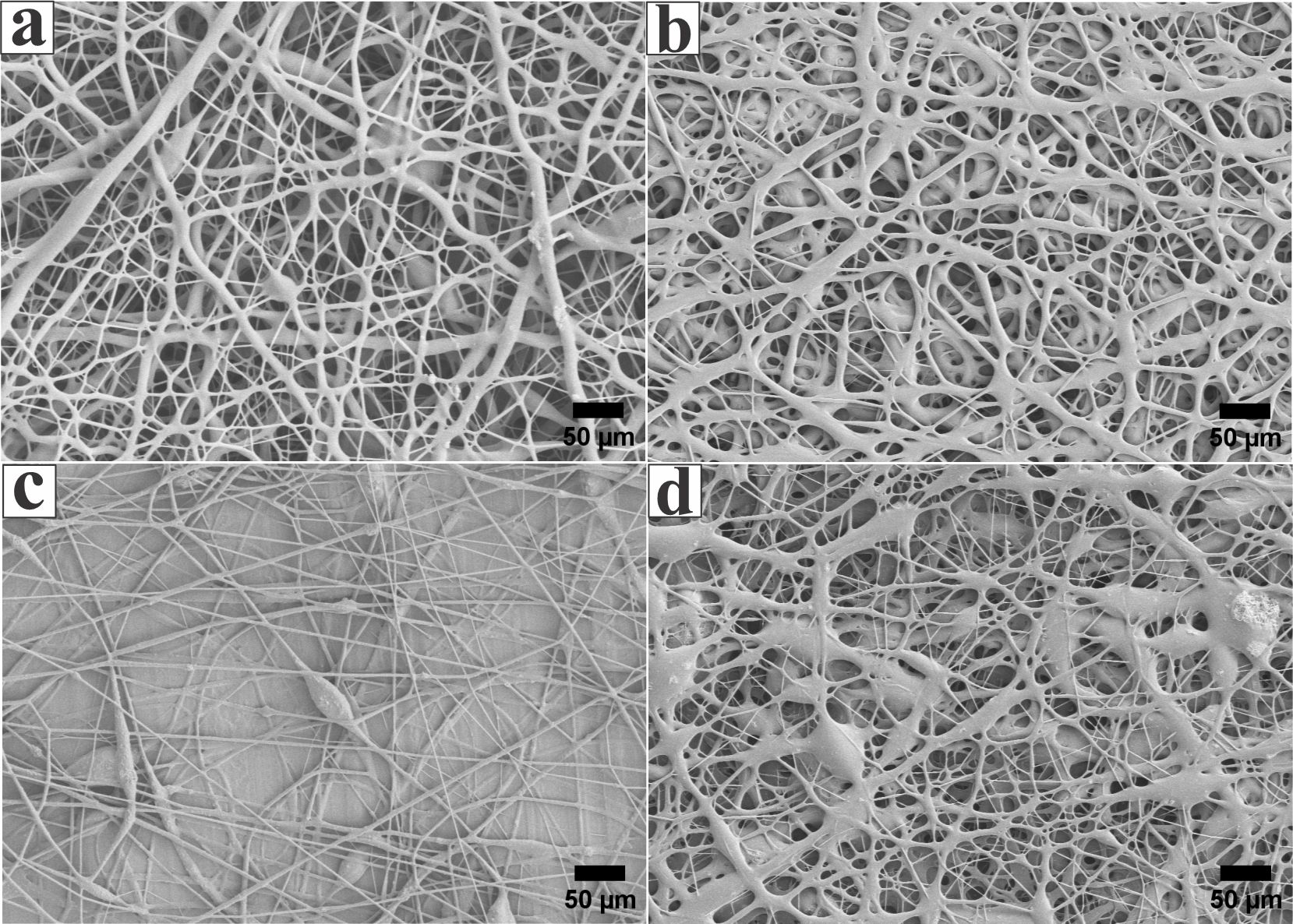


**Fig. S1** SEM images of core-shell nanofiber producibility to optimize the concentration of polymers and HA. (a)10%PCL-18%P, (b) 12%PCL-18%P, (c) 15%PCL-18%P-HA6%, and (d) 15%PCL-18%P-HA8%


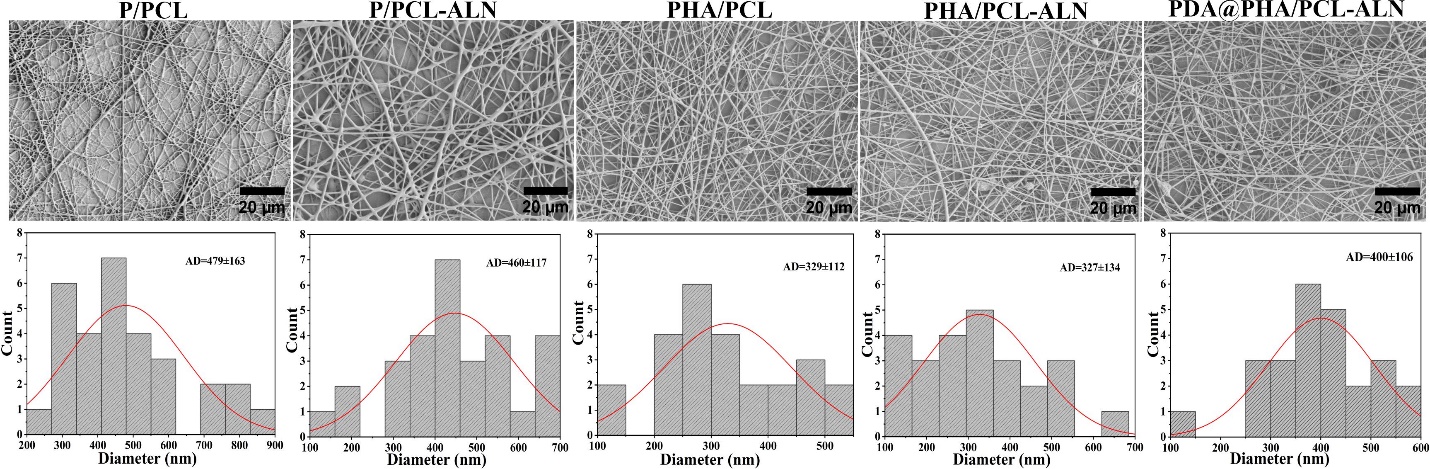


**Fig. S2** SEM images of different core-shell nanofibers with low magnification images and corresponding diameter distribution

**
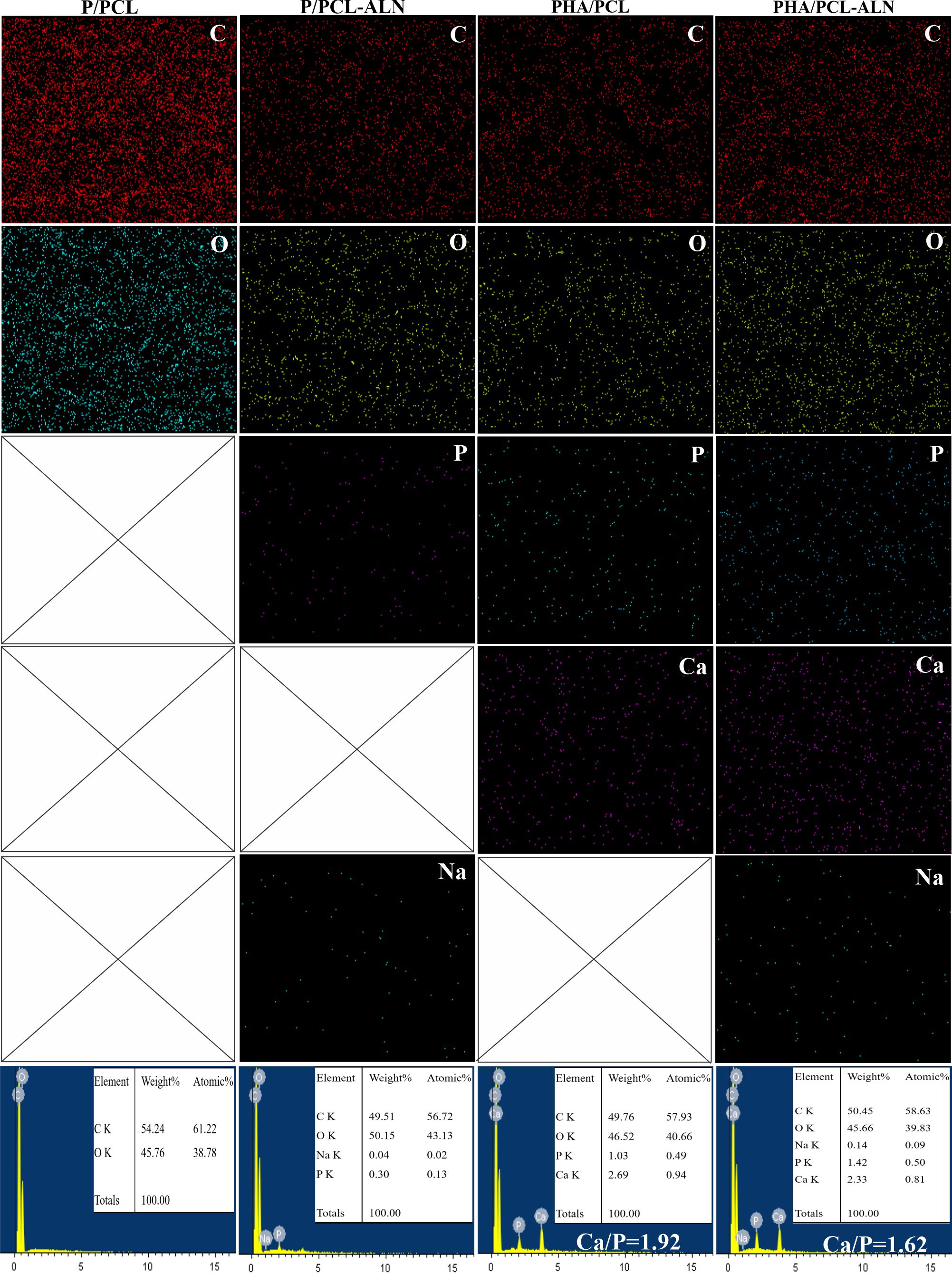
Fig. S3** EDS elemental mapping and EDS spectrogram of core-shell nanofibers P/PCL, P/PCL-ALN, PHA/PCL, PHA/PCL-ALN, and PDA@PHA/PCL-ALN


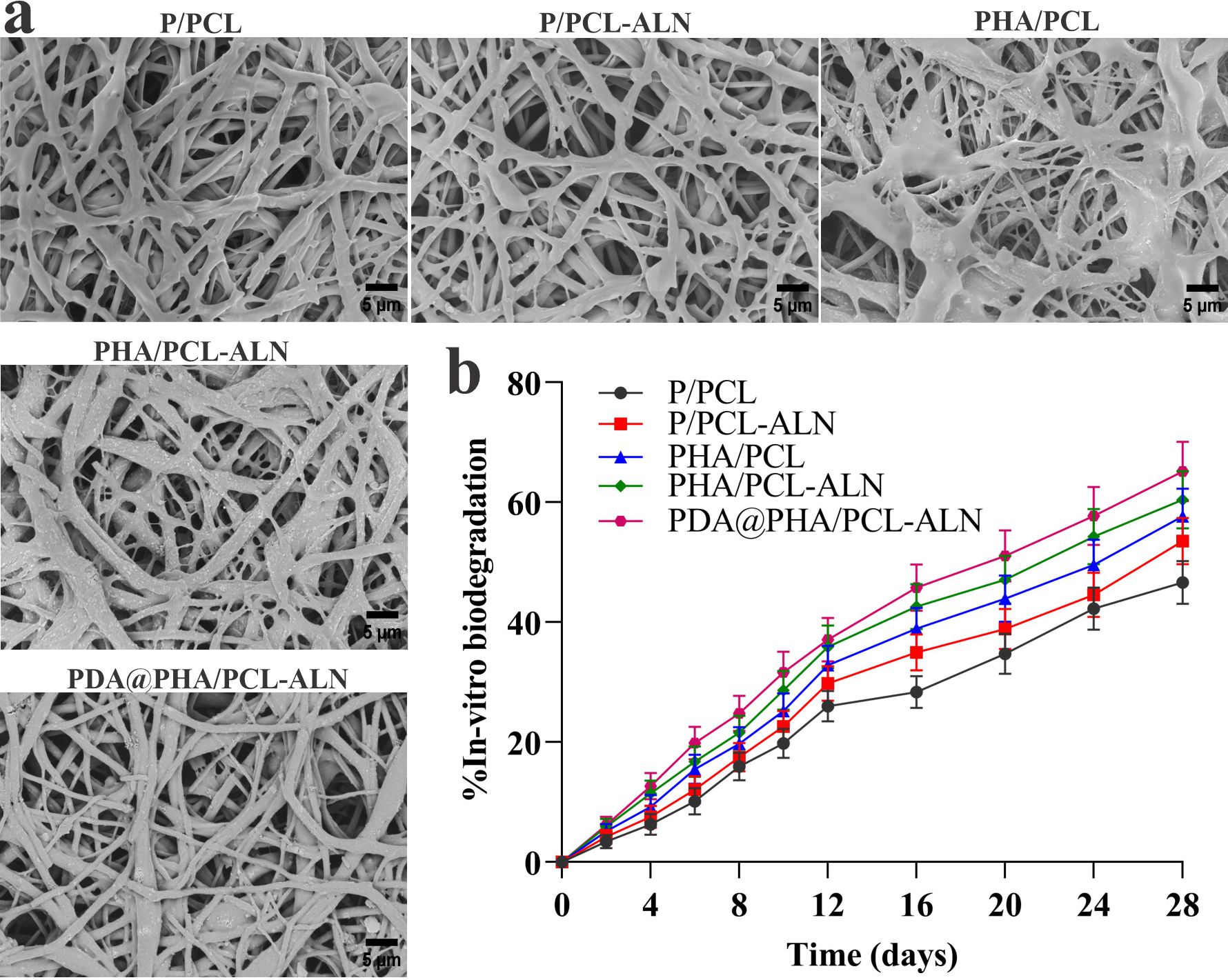


**Fig. S4** *In vitro* biodegradation behavior of different core-shell nanofibrous scaffolds. (a) The SEM images of P/PCL, P/PCL-ALN, PHA/PCL, PHA/PCL-ALN and PDA@PHA/PCL-ALN fibrous scaffolds after degradation for 12 days; (b) Corresponding weight loss of different nanofibrous scaffolds


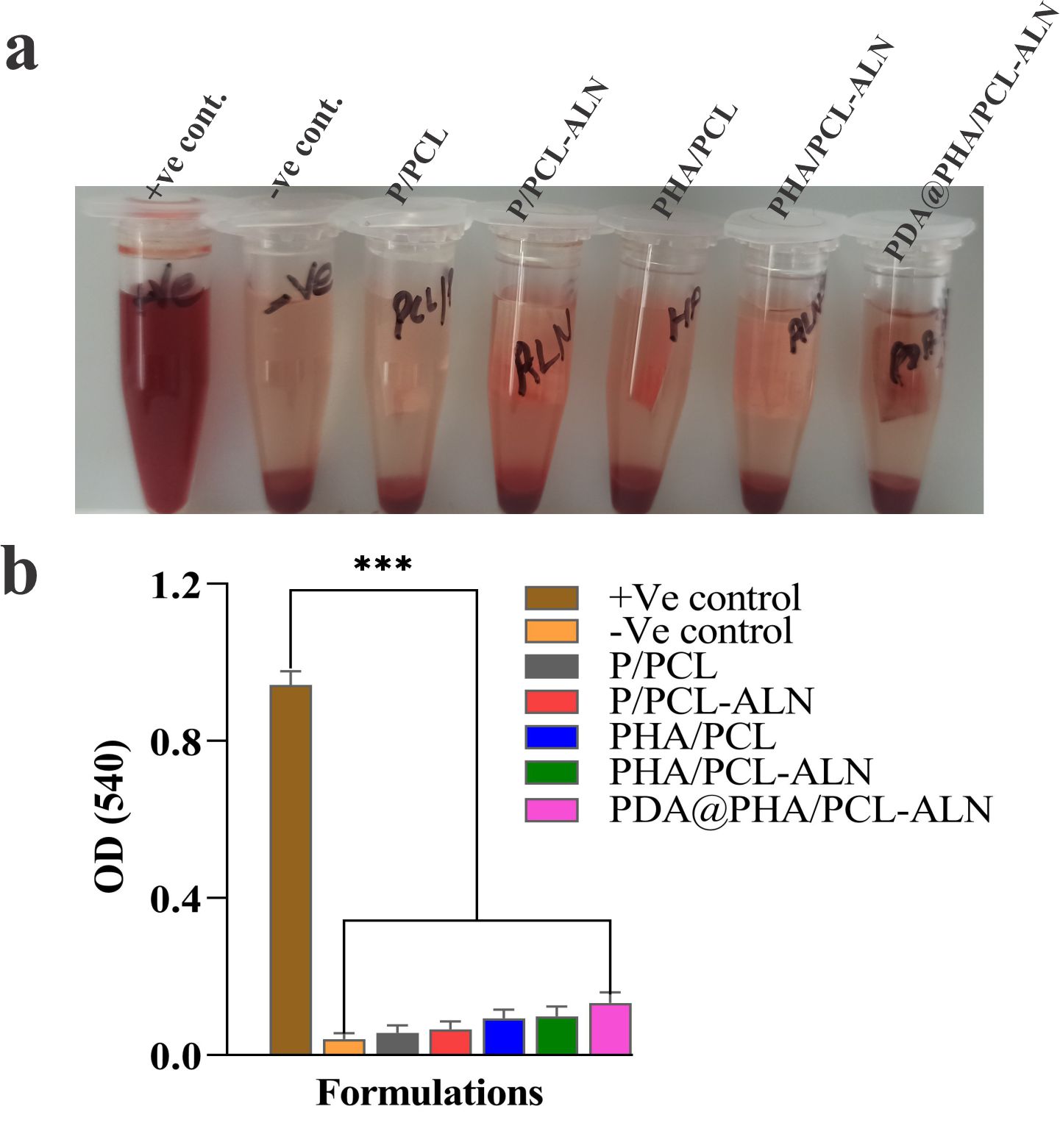


**Fig. S5** Hemolysis study and the effect of scaffold on the lysis RBCs. (a) The image of intact RBCs after 3 hours of incubation with the control group and core-shell nanofiber groups; (b) The histogram demonstrated the absorbance value of +Ve control, -Ve control, P/PCL, P/PCL-ALN, PHA/PCL, PHA/PCL-ALN, and PDA@PHA/PCL-ALN at 540 nm. It is confirmed from the above figure that the created core-shell scaffolds are non-hemolytic and exhibited significantly low level of RBCs disruption suitable for BTR applications


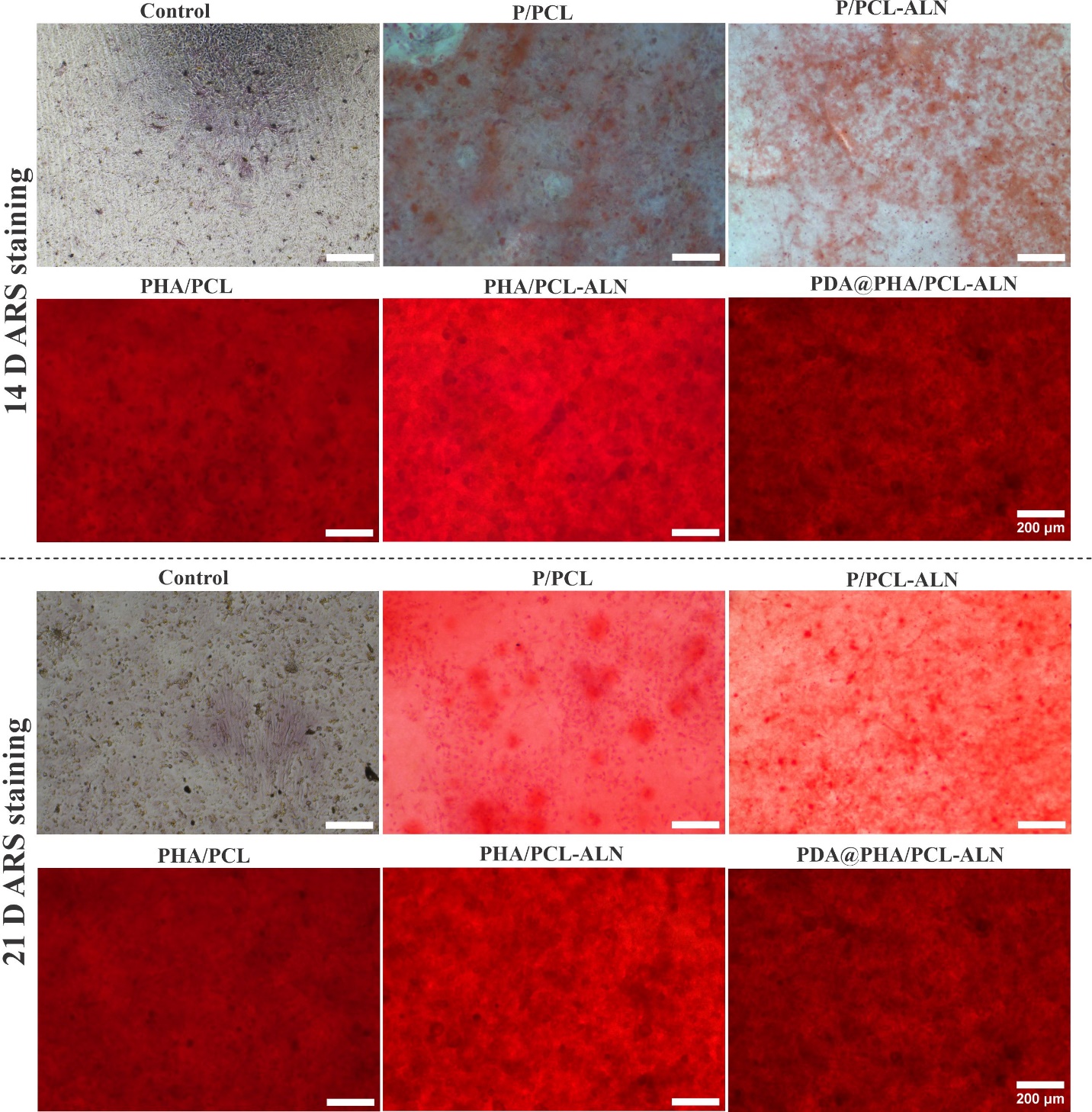


**Fig. S6** ARS staining images of MC3T3-E1 cells cultivated on different core-shell nanofibers, after 14 and 21 days


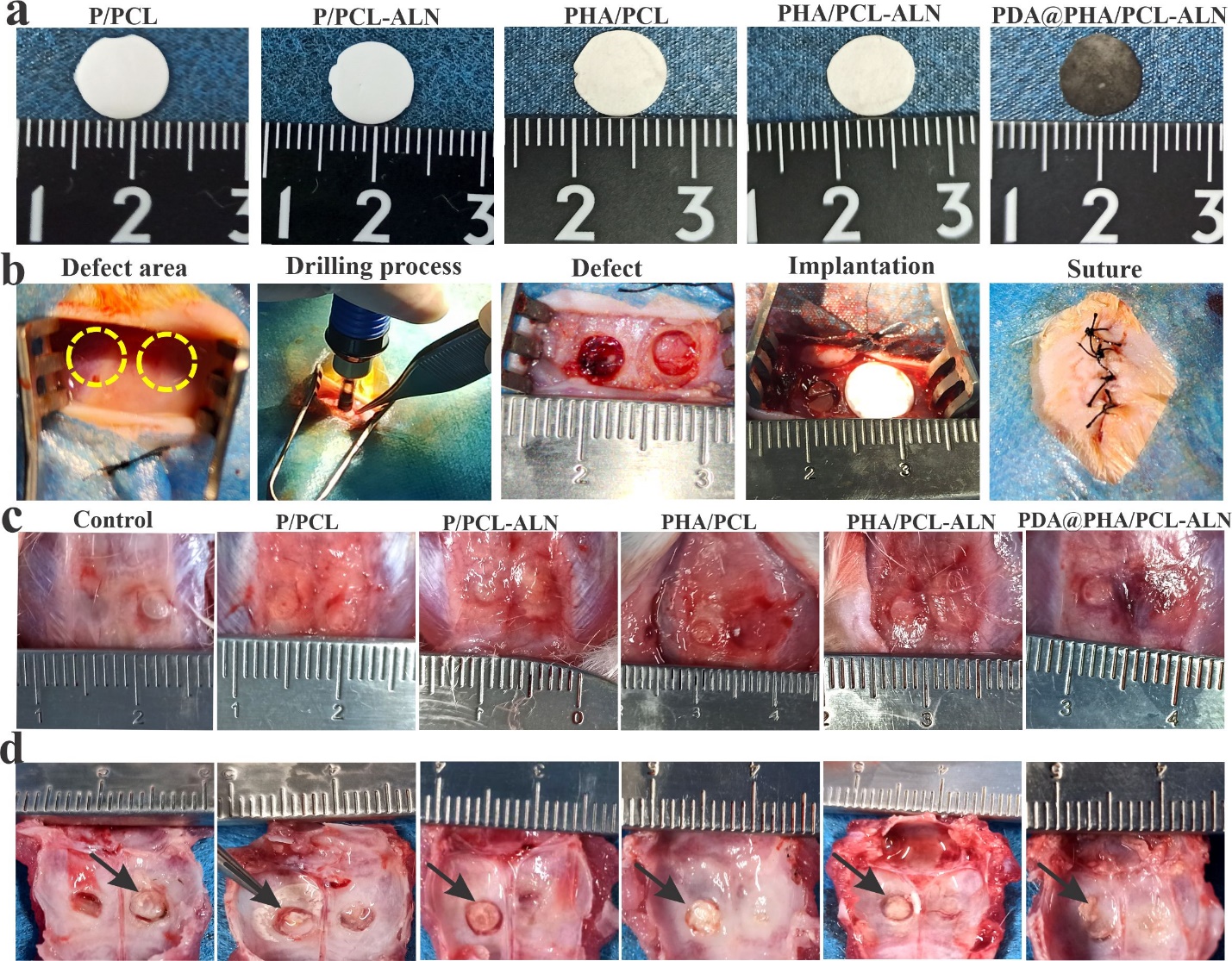


**Fig. S7** (a) Photographs of the different functionalized nanofibers; (b) The surgical procedure during scaffold implantation. The yellow dotted lines indicate the boundary of the defect (4 mm in diameter); (c) A macroscopic front, and (d) back view of the skull bone samples after 12 weeks’ implantation. Scaffolds implanted on the right side of skull defects with bone granules and on the left side of skull defect only bone granules present. Black arrow represents bone granules (Autograft)


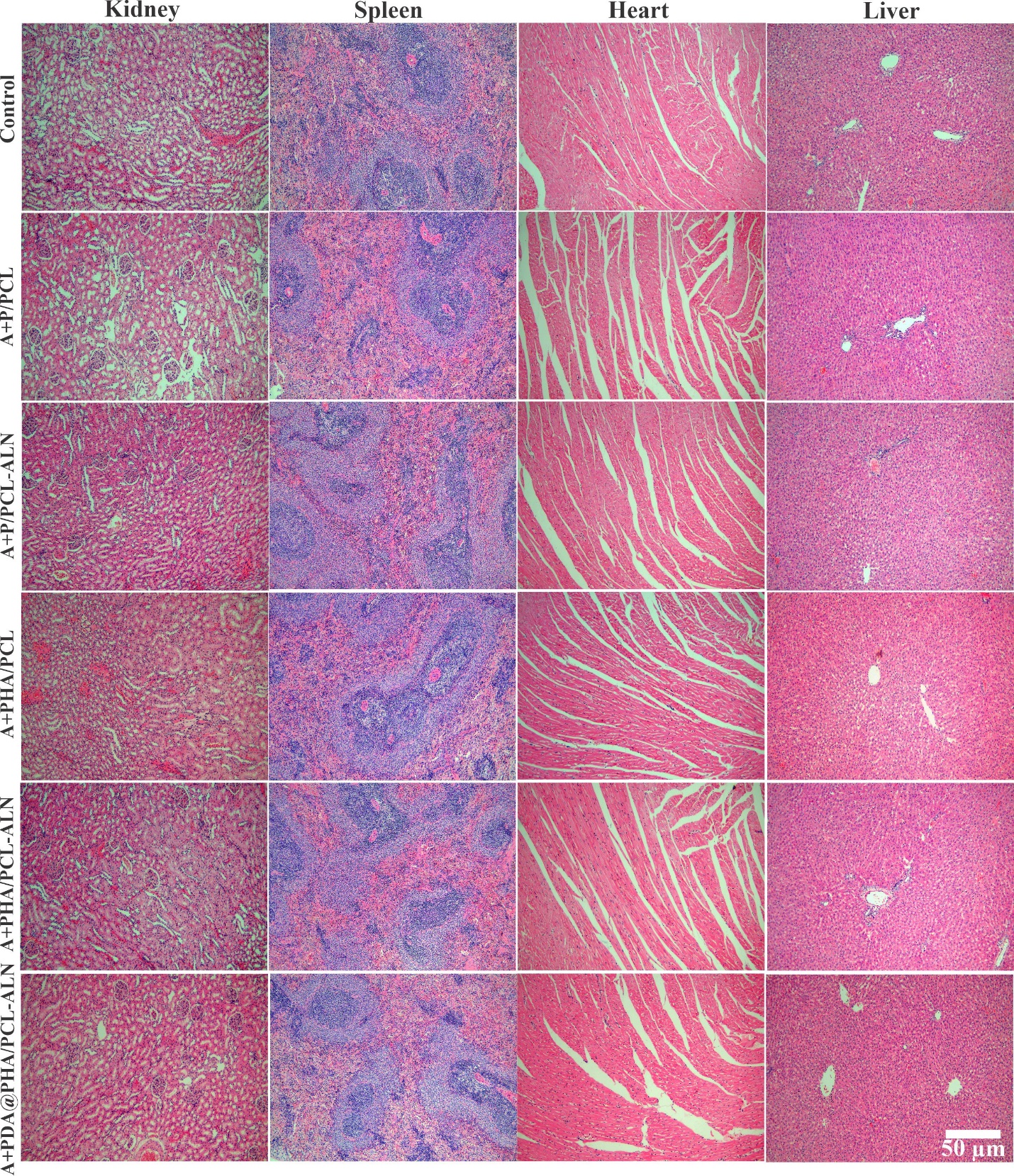


**Fig. S8** H&E-stained microscopic images from histological sections of different organs 12 weeks after scaffold implantation. The H&E-stained images indicated the non-significant changes in kidney, spleen, heart and liver organs underwent the treatment with P/PCL, P/PCL-ALN, PHA/PCL, PHA/PCL-ALN, and PDA@PHA/PCL-ALN core-shell nanofibers

**References**

1. He, Z.; Liu, S.; Li, Z.; Xu, J.; Liu, Y.; Luo, E. Coaxial TP/APR Electrospun Nanofibers for Programmed Controlling Inflammation and Promoting Bone Regeneration in Periodontitis-Related Alveolar Bone Defect Models. *Mater. Today Bio* **2022**, *16*, doi:10.1016/j.mtbio.2022.100438.

2. Pandey, G.; Pandey, P.; Arya, D.K.; Kanaujiya, S.; Deepak Kapoor, D.; Gupta, R.K.; Ranjan, S.; Chidambaram, K.; Manickam, B.; Rajinikanth, P.S. Multilayered Nanofibrous Scaffold of Polyvinyl Alcohol/Gelatin/Poly (Lactic-Co-Glycolic Acid) Enriched with Hemostatic/Antibacterial Agents for Rapid Acute Hemostatic Wound Healing. *Int. J. Pharm.* **2023**, *638*, doi:10.1016/j.ijpharm.2023.122918.

3. Yadav, S.; Arya, D.K.; Pandey, P.; Anand, S.; Gautam, A.K.; Ranjan, S.; Saraf, S.A.; Rajamanickam, V.M.; Singh, S.; Chidambaram, K.; et al. ECM Mimicking Biodegradable Nanofibrous Scaffold Enriched with Curcumin/ZnO to Accelerate Diabetic Wound Healing via Multifunctional Bioactivity. *Int. J. Nanomedicine* **2022**, *17*, 6843–6859, doi:10.2147/IJN.S388264.

4. Agarwal, Y.; Rajinikanth, P.S.; Ranjan, S.; Tiwari, U.; Balasubramnaiam, J.; Pandey, P.; Arya, D.K.; Anand, S.; Deepak, P. Curcumin Loaded Polycaprolactone-/Polyvinyl Alcohol-Silk Fibroin Based Electrospun Nanofibrous Mat for Rapid Healing of Diabetic Wound: An in-Vitro and in-Vivo Studies. *Int. J. Biol. Macromol.* **2021**, *176*, 376–386, doi:10.1016/j.ijbiomac.2021.02.025.

5. Wang, J.; Shao, L.; Wu, X.; Liu, C.; Ni, S.; Dai, T.; Liu, H.; Zhao, H. Electrospun Sandwich Mesh Structures Loaded with Naringenin and Vitamin K2 Polycaprolactone/Gelatin Nanofibers Synergistically Promote Bone Regeneration. *Mater. Today Bio* **2023**, *23*, doi:10.1016/j.mtbio.2023.100794.
